# A Cell-Based Assay for Measuring Endogenous BcrAbl Kinase Activity and Inhibitor Resistance

**DOI:** 10.1371/journal.pone.0161748

**Published:** 2016-09-06

**Authors:** Steven B. Ouellette, Brett M. Noel, Laurie L. Parker

**Affiliations:** Department of Medicinal Chemistry and Molecular Pharmacology, College of Pharmacy, Purdue Center for Cancer Research, Purdue University, West Lafayette, Indiana, United States of America; University of Sydney, AUSTRALIA

## Abstract

Kinase enzymes are an important class of drug targets, particularly in cancer. Cell-based kinase assays are needed to understand how potential kinase inhibitors act on their targets in a physiologically relevant context. Current cell-based kinase assays rely on antibody-based detection of endogenous substrates, inaccurate disease models, or indirect measurements of drug action. Here we expand on previous work from our lab to introduce a 96-well plate compatible approach for measuring cell-based kinase activity in disease-relevant human chronic myeloid leukemia cell lines using an exogenously added, multi-functional peptide substrate. Our cellular models natively express the BcrAbl oncogene and are either sensitive or have acquired resistance to well-characterized BcrAbl tyrosine kinase inhibitors. This approach measures IC_50_ values comparable to established methods of assessing drug potency, and its robustness indicates that it can be employed in drug discovery applications. This medium-throughput assay could bridge the gap between single target focused, high-throughput *in vitro* assays and lower-throughput cell-based follow-up experiments.

## Introduction

Protein tyrosine kinases (PTKs) act as central hubs in cellular signaling that tightly control critical cellular functions such as proliferation, apoptosis, and differentiation. Genetic mutations can alter kinase activity regulation and result in aberrant signaling that promotes disease pathology, most notably cancer [1]. Over 20 tyrosine kinase inhibitors (TKIs) are FDA approved and have varying degrees of clinical success [2]. Despite TKIs often being the best option for patients, heterogeneous response and acquired resistance remain a significant clinical and economic burden. Drug developers are working to overcome these problems by commercializing next generation inhibitors with increased potency, different modes of inhibition, and strategic inhibition of multiple kinases. For example, three generations of BcrAbl kinase inhibitors have been approved for clinical use. Imatinib was the first commercially successful BcrAbl inhibitor, showing an 80% response rate in chronic myeloid leukemia (CML), outperforming the prior therapeutic options and turning CML into a manageable condition [3]. Second generation inhibitors, nilotinib and dasatinib, have increased potency for BcrAbl and have different kinase inhibition profiles than imatinib [4,5]. Other BcrAbl inhibitors, bosutinib and ponatinib, further built on this theme with increased potency and multi-kinase inhibition, and targeting of the T315I gatekeeper mutation, respectively [6,7]—with further introduction of others such as radotinib, now in clinical trials, and many more are in pre-clinical development [8,9].

Kinase activity profiling technologies are essential to identify potential TKIs that target overactive kinases driving disease pathology. Typically, pre-clinical development of TKIs relies on both *in vitro* and cell-based kinase activity assays to select compounds for further development [10–12]. Kinase activity also has an emerging role as a biomarker for predicting response to therapy, including TKIs and pre-operative radiotherapy [13–16], although it is still fairly far away from widespread clinical application in making treatment decisions. *In vitro* assays can be very high-throughput, but are not as biologically informative as cell-based assays. A number of technologies are currently available for cell-based kinase activity assays. Most commercial kinase activity assays for drug development depend on antibody-based detection of endogenous phosphorylation sites as surrogate markers for kinase activity [12]. This strategy takes on many embodiments, including immunoblots, in-cell westerns, homogenous sandwich assays, and high-throughput flow cytometry [11,17–19]. However, site-specific antibodies are expensive to develop and produce, always display some level of non-specificity (which can significantly confound interpretation when trying to analyse an endogenous substrate in complex cellular milieu), and can vary in quality between production lots. Also, the status of endogenous substrate- and autophosphorylation sites is not always representative of the activation state of the kinase itself [20–22]. Changes in the phosphorylation status of an endogenous substrate may take a significant amount of time after addition of TKI due to a combination of intracellular processes (e.g. phosphatase, protein-protein interactions, substrate turnover/synthesis, etc). Because of the baseline level of endogenous substrate phosphorylation, there is a physiological lag time between TKI-mediated BcrAbl kinase inhibition and the reflective change in detectable phosphorylation status of the chosen substrate. Also, apparent BcrAbl inhibition is dependent on which endogenous substrate is measured. For example, Frietsch *et al* show that Tyr-171 on the adaptor protein LASP1 is a bona fide substrate of BcrAbl, and when phosphorylated binds to the SH2 domain of dephosphorylated CrkL [22]. Although both are markers of activity, the differing phosphorylation kinetics and ability to be measured with antibodies/other detection methods could give differing information about levels of Abl kinase activity. Although they may be able to be standardized if a fortuitously reliable antibody is available, these endogenous substrates are highly dependent on site-specific antibody availability. Furthermore, in a clinical setting Frietsch *et al* also show that pCrkL is not always detectable in patient samples, even for those that respond to TKIs [22]. Antibody independent cell-based kinase assays often require genetic manipulation or kinase overexpression systems, affecting the physiological relevance of the assay results [23–25]. Still other methods rely on measuring enzymatic activity from cell lysates, reducing the physiological relevance of the results [23,26]. These limitations have made it difficult to comprehensively measure endogenous tyrosine kinase activity in disease-relevant cellular models, posing limitations in pre-clinical kinase inhibitor development and in translating their use to measuring kinase activity in clinical samples for the purposes of characterizing target-focused response.

Our group develops approaches for measuring cell-based kinase activity assay using multifunctional peptide probes. The peptides contain modular amino acid sequences that each provide a specific function for their interaction with live cells and the kinase of interest (S1 Table). The BcrAbl kinase peptide probe reported by our group has three modular sequences. The “substrate” sequence (EAIYAAPF) binds the kinase domain of BcrAbl and, in the presence of active BcrAbl, is phosphorylated on the central tyrosine residue. The adjacent sequence (APTYSPPPPP) selectively binds the Abl SH3 domain and increases the affinity of the peptide for Abl kinase. The SH3-probe interaction is hypothesized to enhance specific phosphorylation of the substrate by BcrAbl, as implicated in our previous work [27–29] Finally the TAT cell penetrating sequence (RKKRRQRRRLL) facilitates uptake by the cell. We assess BcrAbl activity by measuring phosphorylation of the substrate sequence [27]. The multifunctional peptide substrate’s unique ability to enter many cell types and measure endogenous kinase activity could overcome many of the limitations described above that are associated with the current techniques. Our previous demonstrations of the BcrAbl assay have used mass spectrometry, immunoblot, and ELISA for detection of substrate phosphorylation [21,27–29]. Here we adapt our assay for measuring cell-based BcrAbl kinase activity in a multi-well plate format and improve assay performance. While this detection strategy does rely on an antibody, it does not require any substrate capture antibody and works with the highly robust, generic 4G10 phosphotyrosine antibody for detection, and thus is not dependent on unique substrate or site-specific antibodies—accordingly, the assay protocols reported here would also be compatible with other kinases by simply substituting the biotinylated substrate for a different peptide, without extensive additional development. In the work reported here, we use the method to profile BcrAbl activity in chronic myeloid leukemia cell lines that have been developed to exhibit growth in the presence of moderate concentrations of the kinase inhibitor drugs imatinib, nilotinib and dasatinib, and examine the relationships between BcrAbl kinase activity and cell growth in the presence of each inhibitor.

## Materials and Methods

### Peptide Synthesis

The reporter substrate was synthesized using Fmoc-based solid phase peptide synthesis on a Prelude synthesizer from Protein Technologies (Tucson, AZ). Fmoc-protected amino acid monomers were purchased from Peptides International (Louisville, KY). Fmoc–biotinylated lysine was obtained from Akaal Organics (Long Beach, CA). The photocleavable residue (3-(2-nitrophenyl)-3-aminopropionic acid) was obtained from Lancaster Synthesis and Fmoc-protected by J. Thomas Ippoliti’s lab at the University of St. Thomas (St. Paul, MN). Peptide quality control figures are located in S5–S7 Figs.

### Peptide labeling

The free cysteine residue in the Abl substrate peptide was labeled with Alexa Fluor^**®**^ 488 C_5_ maleimide. The peptide was dissolved to 1 mM in reaction buffer (6M Guanidinium HCl, 100 mM Na_2_PO_4_, 1 mM tris(2-carboxyethyl)phosphine, pH 6.5). 1 mg of Alexa Fluor^**®**^ 488 C_5_ maleimide was dissolved in 20 μL of DMSO and added to the peptide in a 1:1 molar ratio. The reaction was allowed to proceed for 6 hours at room temperature with rocking. After 6 hours 1 μL of 10 M NaOH was added to readjust pH. Completion of the labeling was confirmed by LCMS, and the labeled peptide was desalted, lyophilized, and stored at -20 C until use. Alexa Fluor^**®**^ 488 C_5_ maleimide was obtained from Life Technologies (Carlsbad, CA).

### Cell culture and generation of drug resistant K562 cell lines

The K562 cell line was obtained from ATCC (Rockville, MD) and maintained in Iscove’s Modified Dulbecco’s Media supplemented with 10% fetal bovine serum and 1% penicillin / streptomycin at 37°C in a 5% CO_2_ humidified atmosphere. Resistant K562 cell lines were developed as described previously[30,31]. Briefly, K562 cells were cultured in a tolerated, low dose of imatinib, nilotinib, or dasatinib. Once cells reached 1x10^6^ cells / mL, they were passaged to 0.1x10^6^ cells / mL in media containing an incrementally higher concentration of inhibitor. Increments of inhibitor concentration were determined by observing cell growth inhibition, at which point the concentration was held constant until the proliferation rate was comparable to the parental K562 cell line. This subculturing technique was carried out for 90 days, after which the resistant cell lines were maintained in a final concentration of inhibitor. Final concentrations for K562-IR, K562-NR, and K562-DR cells were 1 μM imatinib, 10 nM nilotinib, and 1 nM dasatinib, respectively. For dose-response studies cells were treated with imatinib (10, 100, 250, 500, 750, 1000, or 10000 nM), nilotinib (0.01, 0.1, 1, 10, 50, 100, or 1000 nM), or dasatinib (0.01, 0.1, 1, 10, 50, 100, or 1000 nM).

### Cell-based kinase assay

K562 cells were seeded at a final volume of 750 μL with the indicated number of cells into 1 mL/well AcroPrep Advance 96 Filter Plates from Pall Corporation (Ann Arbor, MI). The cells were dosed with the indicated tyrosine kinase inhibitor or vehicle control (0.1% (v/v) DMSO) and incubated for 1 hour at 37°C in a 5% CO_2_ humidified atmosphere. Following incubation with TKI, peptide substrate was added at the indicated concentrations and incubated for 5 minutes at 37°C in a 5% CO_2_ humidified atmosphere. Following incubation with peptide, cell media was removed from the filter plate using a vacuum manifold from Pall Corporation, the filter plate was stacked on top of a black Neutravidin™ coated 96-well plate (G-Biosciences, St. Louis, MO) containing 100 μL wash buffer (composed of TBST (50 mM Tris, 150 mM NaCl, 0.05% Tween 20, pH 7.6) with 5% milk protein, US Biological, Salem, MA) per well. In the upper filter plate, 50 μL of lysis buffer (PhosphoSafe™ Extraction Reagent (Novagen) supplemented with protease inhibitor cocktail (Roche) and 4 mM EDTA (to quench kinase activity) was added to each well. The plate stack was incubated on a short orbital plate shaker at 600 RPM at room temperature for 5 minutes. Following lysis buffer incubation, the plate stack was centrifuged at 1500 rcf for 5 minutes to collect lysate in the bottom plate. After lysate collection, the filter plate was removed and the Neutravidin™ plate was incubated with shaking for 1 hour at room temperature. The plate was then washed three times with 200 μL wash buffer. 100 μL of mouse 4G10 antibody (Millipore, 1:12,000 in wash buffer) was added to each well and the plate incubated for 1 hour at room temperature with shaking as described above. Following incubation with 4G10 antibody, 100 μL of rabbit anti-mouse IgG conjugated to HRP (Pierce, 1:1000 in wash buffer) was added to each well and incubated for 1 hour at room temperature with shaking. The plate was then washed three times with wash buffer as described above, followed by two washes with phosphate buffer (50 mM Na_2_PO_4_, pH 7.5). 100 μL of developing reagent (50 mM Na_2_HPO_4_, 2.3 mM H_2_O_2_, 98 μM Amplex Red™ reagent, pH 7.4) was added to each well, incubated for 30 minutes with shaking as described above, and read on a Biotek Synergy4 plate reader using 532 nm excitation and 590 nm emission wavelengths. For dose-response experiments, values at each concentration were represented as percent vehicle control.

### Statistical analysis

Average (Avg) and standard deviation (SD) values were calculated from positive (n = 93) and negative (n = 96) controls. Outliers were defined as those data points that deviated more than 3SD from the mean. Z’ and signal window (SW) values were derived using the Avg and SD values in the following equations [32]:

$$Z^{\prime }=\frac{\left({Avg}_{pos}-{3SD}_{pos})-({Avg}_{neg}+{3SD}_{neg}\right)}{{Avg}_{pos}-{Avg}_{neg}}$$ (1)

$$SW=\frac{\left({Avg}_{pos}-{3SD}_{pos})-({Avg}_{neg}+{3SD}_{neg}\right)}{{SD}_{pos}}$$ (2)

IC_50_ values were derived by fitting a log(inhibitor) vs. response–variable slope (4 parameter) equation with a least squares fit in GraphPad Prism 6. Within the dose-response curve fitting protocol, IC_50_ values for K562 resistant cell lines were compared to the sensitive, parental K562 cell line and statistically assessed using an extra sum-of-squares F test choosing the logIC_50_ as the selected parameter for comparison.

### Growth inhibition Assay

K562 cells were seeded at 10,000 cells per well in 96-well plates and dosed with the indicated TKI concentrations or vehicle control (0.1% DMSO). Plates were incubated for four days at 37°C in a 5% CO_2_ humidified atmosphere. Following incubation, XTT reagent (ATCC) was added according to manufacturer’s protocol and plates were incubated at 37°C in a 5% CO_2_ humidified atmosphere for 3–4 hours. Specific absorbance was read on a Biotek Synergy4 plate reader at 475 nm and non-specific absorbance read at 660 nm. Absorbance values at 660 nm were subtracted from 475 nm absorbance values, and represented as percent vehicle control.

### Immunodetection

K562, K562-IR, K562-NR, and K562-DR cells were grown to 7.5x10^5^ cells/mL and pelleted by centrifugation. Media was removed, and cells were washed in ice cold PBS. After washing, cells were lysed by a combination of heating and rapid freeze/thaw. Lysis buffer (50mM ammonium bicarbonate pH 8.0, 0.2μM sodium orthovanadate, 4mM EDTA, and Complete protease inhibitor (Roche)) was added to the cell pellet and cells were immediately heated at 95°C for 5 minutes and then snap-frozen in liquid nitrogen, and allowed to thaw on ice for 30 minutes. Cell pellets were vortexed, then centrifuged at 4°C for 20 minutes to remove insoluble cellular debris, and lysate was assayed for protein content. Protein content was normalized to 0.25mg/mL using 50mM ammonium bicarbonate, pH 8.0, and 1 μg total protein for each sample was analysed in duplicate with the antibodies of interest using a WES Simple Western system (Protein Simple) according to the manufacturer’s instructions. The samples were separated for 25 minutes at 375 volts and washed with the antibody diluent for 5 minutes before probing with primary and secondary antibodies for 30 minutes respectively. Data for both replicates and run files (.cbz format) comprising the instrument protocol and results are provided in S1 File.

### Supplementary Methods

Supplementary methods are provided in S1 Methods.

## Results and Discussion

### Assay development strategy

**Fig 1:**
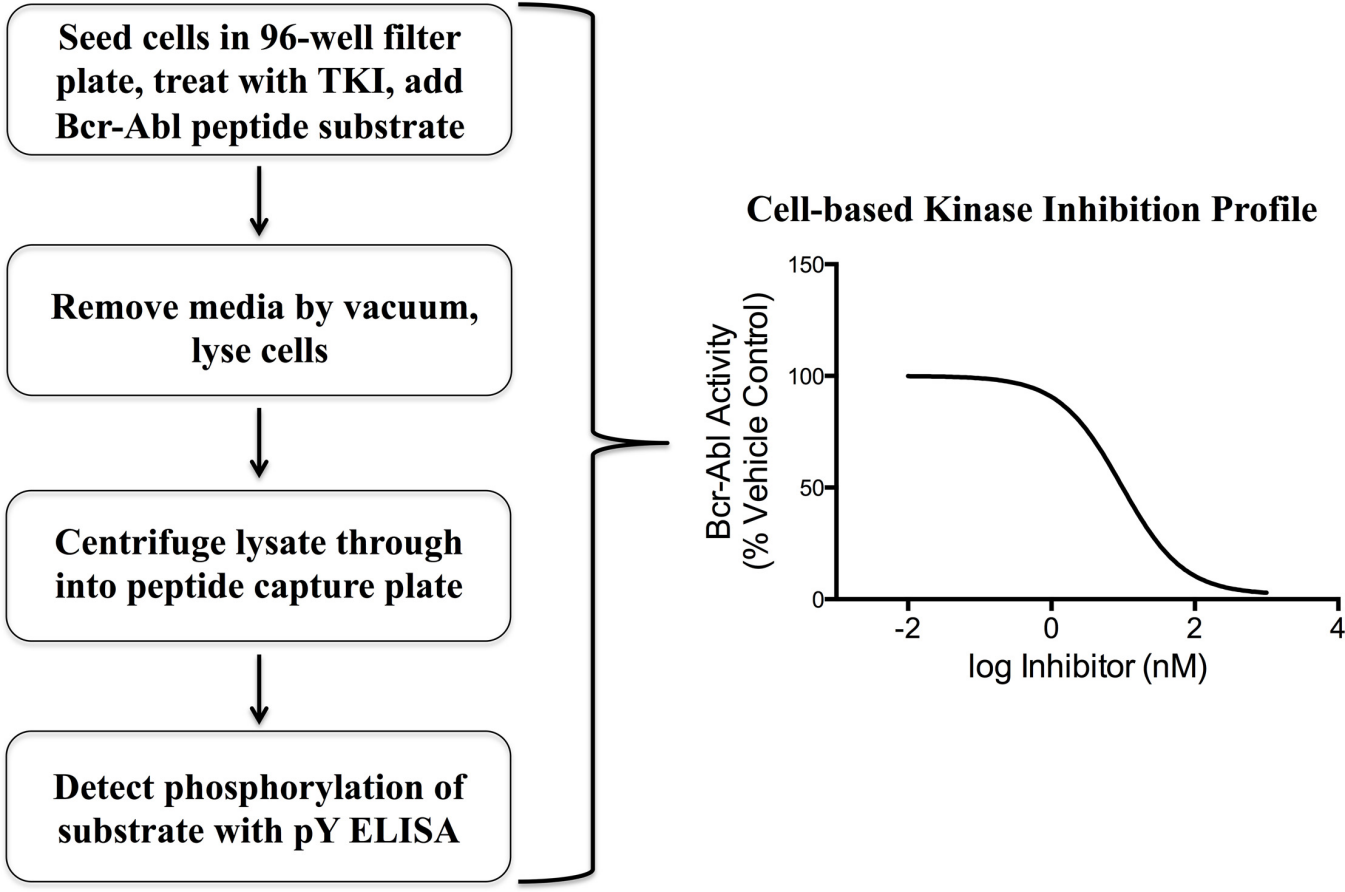
Workflow for cell-based BcrAbl kinase activity assay. Non-adherent K562 cells are seeded into a 1 mL per well capacity, 96-well filter plate, treated with a BcrAbl kinase inhibitor, and incubated with the cell-penetrating BcrAbl substrate peptide. The substrate is taken up into cells and phosphorylated specifically if BcrAbl kinase is active. Following incubation with the substrate, media is removed by vacuum, lysis buffer is added and incubated with the cells. Following lysis, the soluble cell extract is centrifuged through the filter into a Neutravidin™ capture plate and the BcrAbl substrate is immobilized via its biotin tag. Phosphorylation of the peptide is detected through ELISA using a generic anti-phosphotyrosine antibody.

Streamlining the assay for multi-well plates was inspired by a workflow described by Mand and colleagues [33]. The method uses a 96-well filter plate for cell treatment and lysis, followed by ELISA-based detection of substrate phosphorylation (Fig 1). In the study by Mand *et al*, 96-well filter plates were used as a method to prepare K562 cell lysates that were subsequently assayed for Abl kinase activity by incubating the extract with an Abl substrate peptide immobilized on a 96-well, Neutravidin™ coated plate. Phosphorylation of the immobilized substrate was assessed using a well-established pTyr specific antibody in an ELISA-based detection method. Using this strategy, they were able to achieve a medium-throughput assay for measuring Abl kinase activity in cell lysates. While kinase activity in cell lysate provides a more biologically relevant context than purified kinases, it still lacks many important factors within the environment of a living cell, such as kinase and phosphatase localization, intracellular ATP concentrations, and drug transport dynamics. An important aspect of the approach in this report is that the assay signal is representative of intracellular kinase activity in the presence of live cell dynamics. This is achieved by adding the multi-functional peptide substrate to live cells, where it is internalized and phosphorylated prior to cell lysis. This provides biological relevance that is lost when examining kinase activity outside of a living cell.

### Assay workflow standardization

**Fig 2:**
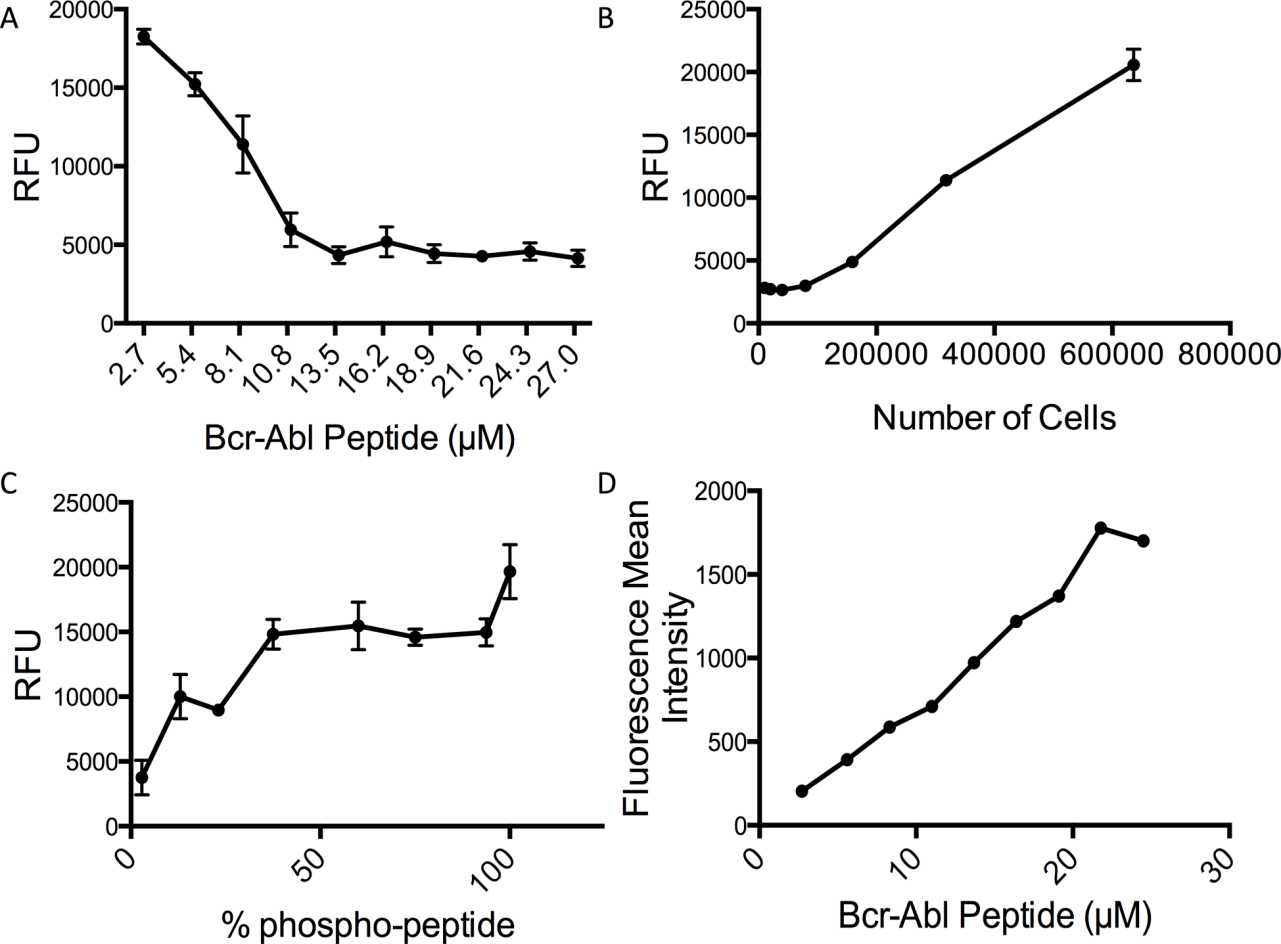
Standardization of peptide concentration and cell number BcrAbl kinase activity assay. Peptide concentration and cell density were standardized for the cell-based BcrAbl kinase activity assay. BcrAbl kinase activity signal reported as relative fluorescent units (RFU, A-C) (A) Cells were exposed to a range of peptide concentrations (2.5–25 μM) to determine the effect on variation and signal to background ratio (n = 8, error bars represent SEM). (B) Cell density was varied using untreated K562 cells to determine the minimum number of cells to perform the assay. (n = 3, error bars present for all data points and represent SEM) (C) Phosphorylated and unphosphorylated BcrAbl substrates were mixed in different ratios, spiked into quenched K562 lysate, and added to the Neutravidin™ coated plate. Each condition contained 30 pmol of phosphorylated substrate and the amount of unphosphorylated substrate was varied to change the ratio (n = 3, error bars present for all data points and represent SEM). (D) K562 cells were exposed to different concentrations of the BcrAbl substrate tagged with Alexa Fluor^®^ 488 and cellular uptake was assessed using flow cytometry. The mean fluorescence intensity is plotted as a function of substrate concentration.

Our first aim was to show that the streamlined assay protocol could be reliably reproduced. Previous demonstrations of our lab’s cell-based Abl activity assay had coefficients of variation (CVs) ranging from 20–40% [29]. An acceptable CV for cell-based analysis in drug discovery assays should be at or below 20% [32]. To determine if S:B and CV are a function of substrate concentration, we varied the peptide concentration from 2.7 μM to 27 μM in our 96-well plate workflow (Fig 2A). We found that the lowest CV occurred at the lowest substrate concentration tested (S1 Fig), and that this correlated with higher signal to noise ratios (S2 Fig). These results indicate that phosphorylated peptide signal intensity decreases substantially with peptide concentrations above 2.7 μM, reaching a low around 11 μM and remaining constant for all greater concentrations. Based on performance, 2.5 μM peptide substrate was used in all future assays. We determined the lower limit cell density to use in the assay by varying the number of cells per well (Fig 2B). We found that cell densities less than 0.5E6 cells per mL gave unacceptable S:B, and chose to use 750,000 cells per well (10^6^ cells/mL) to keep the most robust signal. Cell density is an important factor that has consequences for assay volume because BcrAbl signaling is reduced when K562 cells exceed (10^6^ cells/mL). In order to decrease assay volume with the chosen 750,000 cells per well, the cell density would have to surpass 10^6^ cells/mL. While this number of cells per well exceeds the typical number of cells for a 96-well plate format, K562 cells are non-adherent and can be seeded in the 1 mL capacity 96-well filter plate at a density that does not impact intracellular signaling.

The highest S:B and acceptable CVs were found at the lowest peptide concentrations tested. We hypothesized that higher concentrations of substrate might result in a lower ratio of phosphorylated substrate to unphosphorylated substrate due to an excess of substrate that the cell internalizes but does not phosphorylate for the duration of the assay. An excess of unphosphorylated peptide could dilute the signal from phosphorylated peptide by out-competing phosphorylated peptide bound to the capture plate, resulting in a lower pTyr signal in the ELISA and reducing the S:B. The predicted binding capacity of each well in the Neutravidin™ capture plates used in this study was 15 pmol. To demonstrate the signal dilution pattern we looked at the effect of different ratios of phosphorylated (30 pmol) to unphosphorylated (increasing amounts) peptides (Fig 2C). As expected, the results show that in the presence of a saturating amount of phosphorylated peptide, adding more unphosphorylated peptide to the well reduces the phospho-peptide signal. Next, we assessed whether changes in cellular uptake could be contributing to the peptide concentration dependent changes in S:B. We exposed K562 cells to the BcrAbl peptide labeled with Alexa Fluor^**®**^ 488 C_5_ maleimide (Life Technologies) at the same range of concentrations used for peptide concentration optimization (2.7 μM to 27 μM), and measured relative peptide uptake using flow cytometry. While presence of the fluorophore can potentially change the peptide’s overall charge and hydrophobicity, thus impacting uptake kinetics, it has been established that the TAT sequence efficiently shuttles cargo labeled with this and similar fluorophores into cells. The results show that each concentration of peptide was associated with a significant rightward shift in the cell population compared to control. A multimodal distribution was not observed for any of the conditions, implying that most of the cells internalized the peptide regardless of concentration (S2 Fig). We found that the mean intensity of substrate associated with the cell population increased from 2.7 μM to 21.6 μM (Fig 2D), whereas the S:B exhibited an inverse change over the same range of concentrations. Another possible explanation for the peptide concentration-dependent effect on S:B is that higher concentrations of substrate disrupt the accessibility of the peptide to BcrAbl, or reduce kinase activity—therefore, the effect of peptide concentration on substrate localization and intracellular BcrAbl signaling was assessed. Substrate localization was visualized with fluorescence microscopy and showed little difference over the range of concentrations, suggesting that localization cannot account for the reduction of S:B (S3 Fig). We measured CrkL phosphorylation as a surrogate marker for endogenous BcrAbl activity after incubation of increasing concentrations of BcrAbl substrate with K562 cells. No change in CrkL phosphorylation was observed, so we conclude that substrate mediated changes to BcrAbl kinase activity do not account for the reduction in S:B (S4 Fig). The flow cytometry results show that the amount of substrate internalized increases proportionally with higher exposure concentrations, implying that the intracellular concentration increases. If there is more intracellular substrate than can be phosphorylated in the given incubation time, then the ratio of unphosphorylated to phosphorylated substrate will increase with the cell exposure concentration, and will translate to a diluted signal. Accordingly, it is important to characterize these factors for future implementation of this assay in other laboratories that may be using capture plates from different sources, as the binding capacity between manufacturers can vary.

### Reproducibility characterization

**Fig 3:**
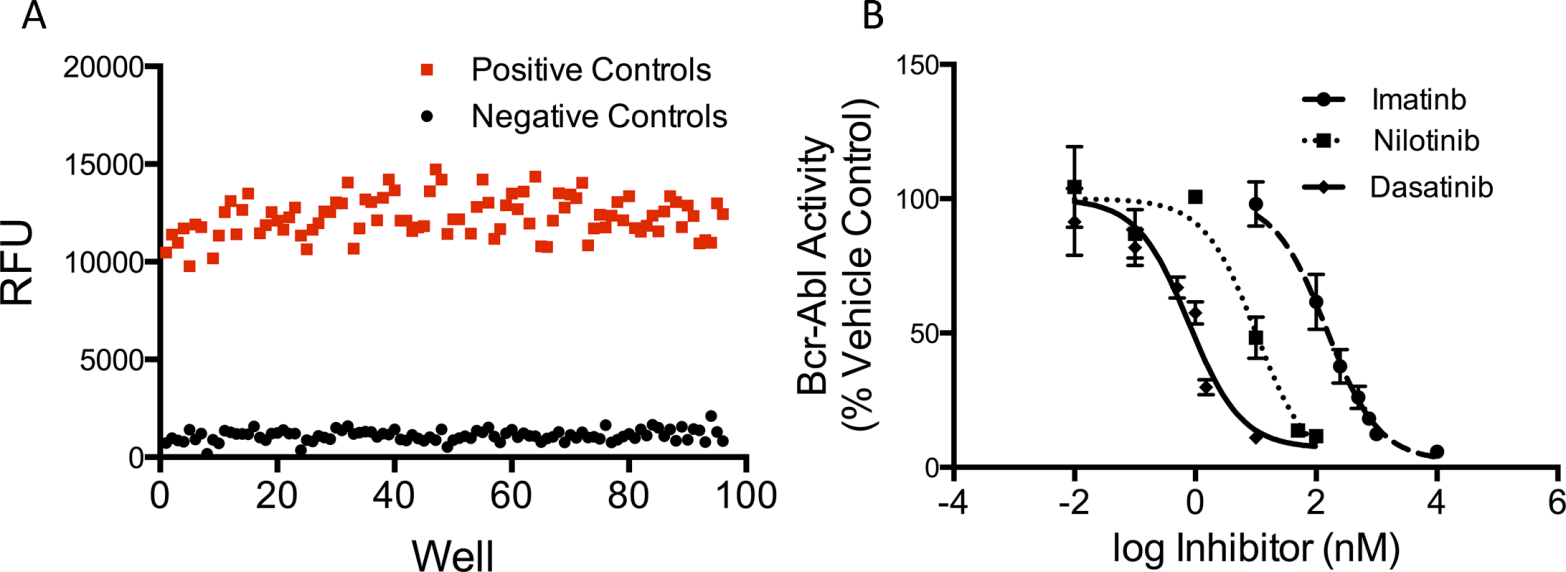
Characterization of assay reproducibility and validation using well-known BcrAbl inhibitors. (A) Positive (n = 93) and negative (n = 96) controls (positive control: maximal phosphorylation in the cell-based kinase activity assay; negative control: corresponding maximal inhibition by pre-incubation of cells with imatinib, 10 μM, followed by cell-based kinase activity assay) were performed in parallel using the standardized workflow and the values were plotted to visualize separation of the signal intensities. BcrAbl activity is reported as relative fluorescent units (RFU) (B) BcrAbl kinase activity dose-response curves in K562 cells using well-characterized BcrAbl inhibitors. Cells were treated with 7 concentrations of either imatinib, nilotinib, or dasatinib for 1 hour prior to addition of the BcrAbl peptide and processed for kinase activity analysis. (n = 3, error bars represent SEM).

To characterize assay reproducibility and assess potential throughput, we determined the signal distributions for positive and negative controls that define the maximum cell-based BcrAbl kinase activity and fully inhibited cell-based BcrAbl kinase activity. Two 96-well filter plates were seeded with K562 cells, and imatinib (10 μM) was added in a checkerboard pattern. The cell-based kinase assay was performed as described in the Materials and Methods. Wells that contained imatinib served as the negative controls (n = 96) and wells without imatinib were positive control wells (n = 93). Z’-factor, signal window, coefficient of variation, and outlier rates were calculated for the positive and negative signal distributions and used to assess the quality of the assay (Fig 3A). Z’-factors greater than 0.5 and signal windows greater than 2 are generally considered suitable for high-throughput screening [32,34]—albeit other practical factors may come into play for actual high-throughput implementation of the overall protocol. Importantly, nearly 100 replicates of each control were performed to clearly define the signal distributions. From these controls we calculated a Z’-factor of 0.64 and a signal window of 7.08. Based on this analysis, the assay is, in principle, suitable for high-throughput screening, even though the number and type of steps in the protocol mean that it would be more practical for up to medium-throughput applications. Over all replicates, the coefficients of variation for positive (CV_pos_) and negative controls (CV_neg_) were 0.08 and 0.26, respectively. Generally, cell-based assays are considered sufficient for high-throughput applications when the CVs for controls are less than 0.2. However, it is not uncommon for CV_neg_ to exceed the 0.2 requirement. In this case, an alternate acceptance criterion is for the standard deviation of the negative controls to be less than the standard deviation of the positive controls, and this was the case for our assay [32]. Finally, outlier analysis indicated that positive and negative controls have outlier rates of 3% and 1%, respectively. The statistical assessment, combined with the automatable nature of the assay workflow, suggests that the assay could be suitable for screening applications. However, the relatively laborious handling steps (e.g. centrifugation and washes) make this assay better suited for detailed characterization of compounds in medium-throughput during the hit to lead phase of drug discovery, rather than primary or secondary screening.

### Validation with well-characterized BcrAbl inhibitors

Next we derived IC_50_ values from our cell-based kinase inhibition assay and compared the values to growth inhibition IC_50_ values reported in the literature. We used three well-characterized, clinically approved inhibitors that target BcrAbl: imatinib, dasatinib, and nilotinib. The kinase activity IC_50_ values for imatinib, nilotinib, and dasatinib were 150 nM (95% CI, 100–223 nM), 10 nM (95% CI, 4–25 nM), and 1 nM (95% CI, 0.5–1.2 nM), respectively (Fig 3B). These values were within 10-fold of the biochemical and cytotoxicity IC_50_ values reported in the literature, and comparable to IC_50_ values for the same TKIs using K562 cell lysates [33,35] However, activity- and cytotoxic-based inhibition assays should be used as complementary approaches for measuring drug action. Cytotoxicity (i.e. growth inhibition) is a measure of how the cellular system responds as a whole to the TKI, and reflects a cumulative phenotypic effect resulting from inhibition of all the drug’s direct and indirect targets. The BcrAbl activity measurement reports a single parameter in the cell (the targeted kinase’s activity), and how it reacts to an inhibitor. Correlation between the two assays can imply or give support to compound mediated kinase activity inhibition as a mechanism for growth inhibition. We know from reported biochemical binding assays that all of the TKIs we tested bind and inhibit BcrAbl *in vitro* [35]. However, when the compound’s mechanism of action is unknown, it is possible that what we observe as kinase inhibition could be a secondary effect of an alternate action, e.g. inhibition of a kinase-deactivating phosphatase. It is important to keep these considerations in mind when comparing targeted cell-based kinase activity assays to phenotypic screening assays. While recognizing these fundamental differences in the growth inhibition and BcrAbl activity assays demonstrated here, the results indicate that our BcrAbl activity assay generates reliable IC_50_ values for drug-mediated BcrAbl inhibition in the K562 CML cell line.

### Application of the assay to characterize BcrAbl activity in TKI-resistant K562 cell lines

A potential key application for this technology is to perform target-focused characterization of drug resistance phenotypes in disease relevant cell models. Current strategies for characterizing resistance measure cell growth/cytotoxicity (which does not report whether the resistance is on- or off-target). We generated K562 cell lines with acquired TKI resistance through TKI dose escalation, as previously described [30,31,36–39]. This process was carried out for 90 days, after which the TKI concentration was kept constant at the highest achieved level during future culture conditions, to maintain the predominance of resistant cells and prevent reversion to the non-resistant parental line. Following the dose escalation period, cells were considered resistant if they could be cultured in the presence of TKI concentrations that killed the parental culture. Three TKI-resistant cell lines were generated using imatinib, nilotinib, or dasatinib, and designated K562-IR, K562-NR, and K562-DR, respectively. It should be noted that the cell lines in this study are not a comprehensive representation of TKI-resistance in CML. Varying levels of resistance exist, and we chose the 90-day dose escalation period because it allowed resistance to emerge as we defined it above. Other groups have driven K562 cells to greater levels of resistance (i.e. higher TKI concentrations over longer periods of time). Tang *et al*, for example, derived a dasatinib resistant K562 cell line that could grow in the presence of 200 nM dasatinib over a 7-month period [39]. The differences in both the time allowed for resistance to develop and TKI concentration will likely result in alternative mechanisms of resistance.

**Fig 4:**
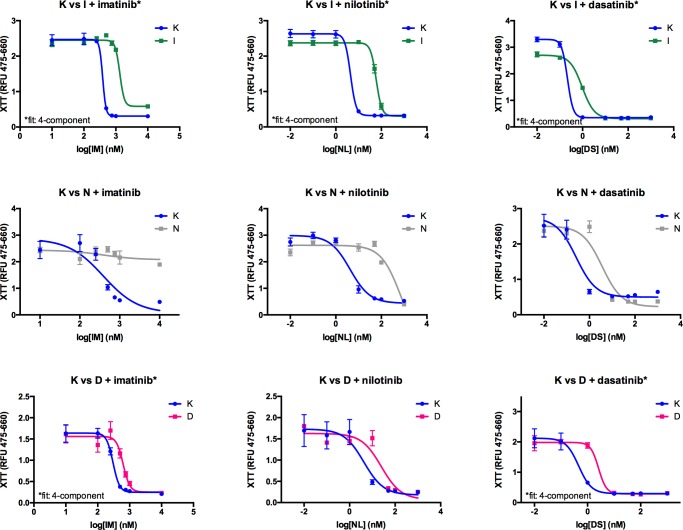
Characterization of TKI-resistance using growth inhibition curves. Dose-response data and fitted curves, and calculated IC_50_ values from growth inhibition curves. TKI-sensitive K562 and the TKI-resistant K562-IR (top row), K562-NR (middle row), and K562-DR (bottom row) cell lines were exposed to the indicated TKI and assayed for mitochondrial function after 72 hours. Data were fitted to non-linear regression models using either three or four parameters, and the best fit to the data was selected for each set of curves. Curves were compared for logIC_50_ using the sum-of-squares F-test. IC_50_ values were derived from these data and used to characterize level of TKI-resistance.

**Fig 5:**
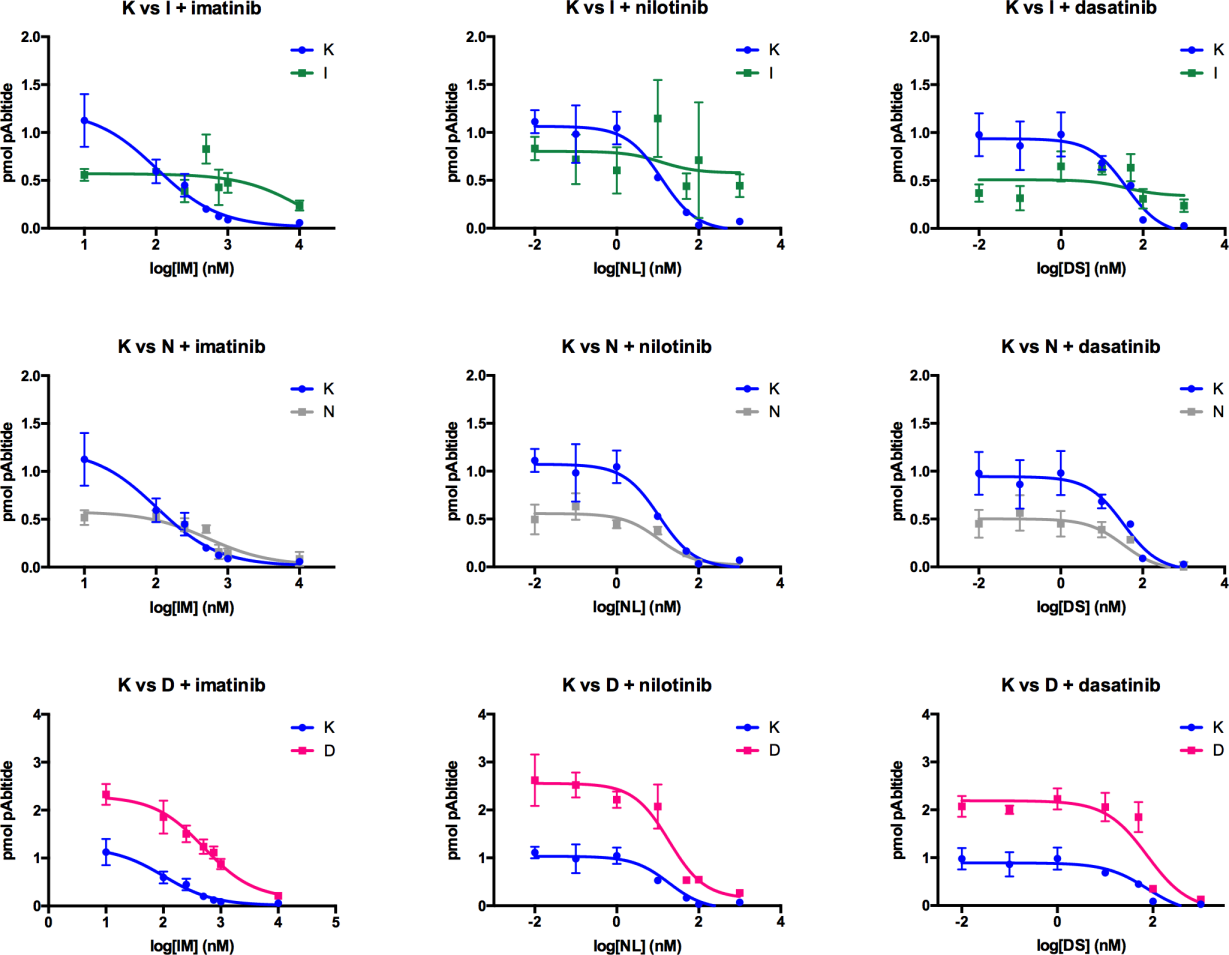
Dose-response data and fitted curves from the BcrAbl activity assay. TKI-sensitive K562 (K) and the TKI-resistant K562-IR (I, top row), K562-NR (N, middle row), and K562-DR (D, bottom row) cell lines were exposed to the indicated TKI and BcrAbl activity was assessed as described in the main text. Data were fitted to a three parameter non-linear regression model. Curves were compared for logIC_50_ using the sum-of-squares F-test. IC_50_ values were derived from these data and used to characterize involvement of Abl activity in TKI-resistance.

**Fig 6:**
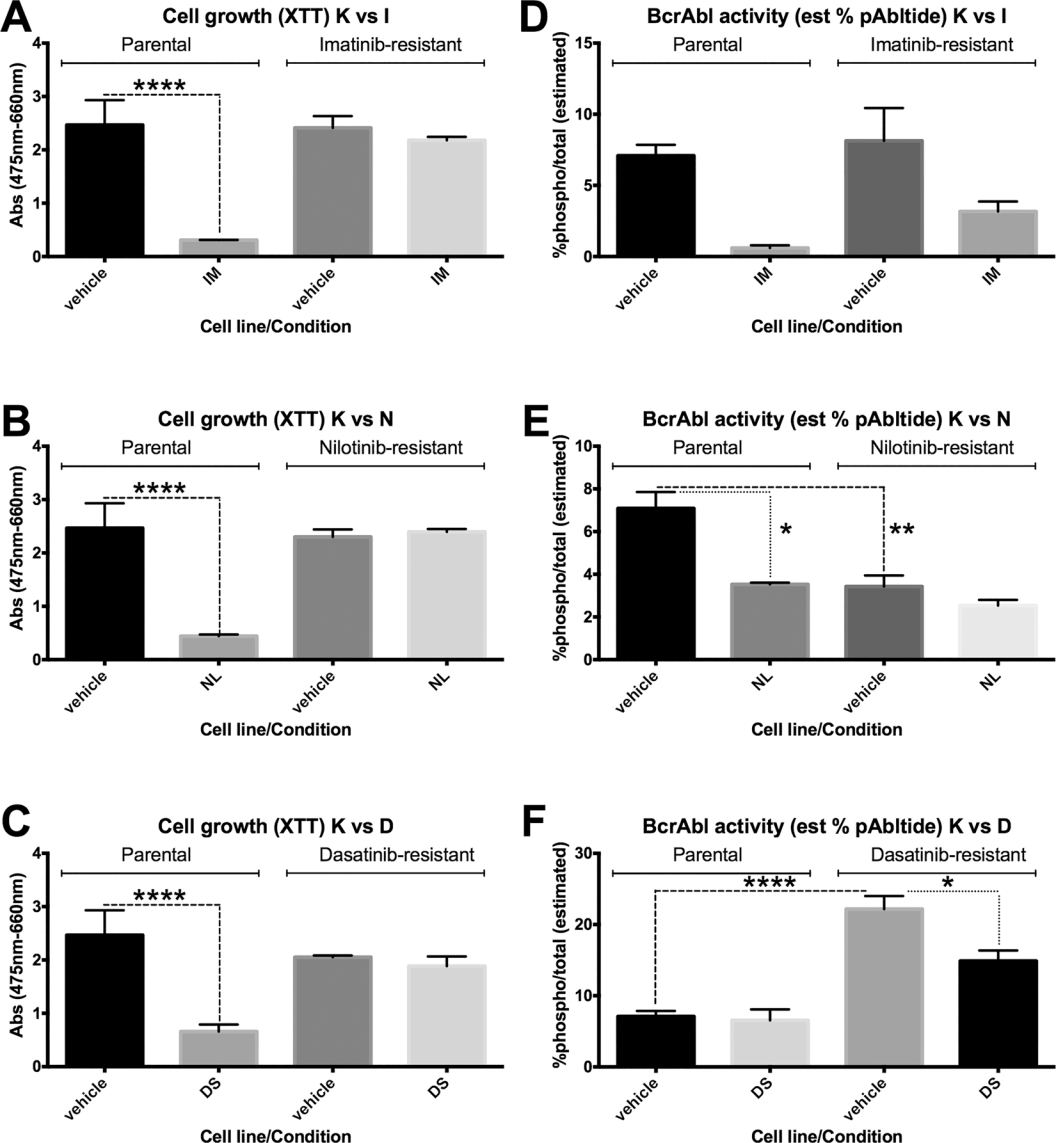
Alternative presentation of selected data from Figs 4 and 5. Data for conditions consistent with the maintenance culture conditions for each cell line were re-plotted separately to show status for growth inhibition (A-C) and BcrAbl activity inhibition (D-F) at relevant concentrations of each drug (1 μM IM, 100 nM NL, 10 nM DS). Comparisons between vehicle and inhibitor treated cells for TKI-sensitive K562 (K/parental, left) and the TKI-resistant K562-IR (I, right) are shown in panels A and D; Parental (left) vs. K562-NR (N, right) are shown in B and E; Parental (left) vs. K562-DR (D, right) are shown in C and F. Cell growth is given as normalized absorbance from the XTT assay. BcrAbl activity is given as estimated % total phosphorylated peptide, based on pmol pAbltide detected (calibrated via standard curve) and the total binding capacity of each well (15 pmol). ANOVA with Tukey’s multiple comparisons test was used to compare means, with significant differences indicated by *, **, or ****.

Drug resistant cell lines were first characterized using a standard strategy by comparing IC_50_ values for growth inhibition relative to the TKI-sensitive K562 parental cell line (Fig 4, Table 1) before assessing BcrAbl activity (Fig 5, Table 1). Loss of potency was observed for each cell line with regard to its applicant TKI. Accurate p-values could not be derived for all cell lines and treatments because the dose-response curves were not always able to be fitted to the same model (which was required for calculating the p value for differences between the curves for the parental vs. resistant cell lines), however it was clear from the growth curves (Fig 4) and from extracted data for the concentrations of each inhibitor used in the culture medium (Fig 6A–6C) that the cells were growing in substantially higher concentrations of inhibitor, and thus resistant. If resistance of each cell line to each inhibitor was due to on-target factors (i.e. changes in the inhibition of BcrAbl activity by the TKI), we reasoned that resistance-related shifts in IC_50_ values for BcrAbl activity inhibition between resistant cell lines and the parental cell line would correlate with shifts in IC_50_ values for growth inhibition. Using the target-focused cell-based BcrAbl activity assay, IC_50_ values were derived in response to imatinib, nilotinib, and dasatinib and (Table 1, Fig 5). BcrAbl activity inhibition IC_50_ values were compared between the sensitive K562 cell line and the resistant derivatives.

**Table 1:** BcrAbl activity and growth inhibition IC_50_ values for TKI resistant cell lines, shifted relative to parental K562 cells.

Cell Line + TKI	Growth Inhibition IC_50_ (95% CI) (nM)	P-value (difference, K562 vs K562-XR)	BcrAbl Activity IC_50_ (95% CI) (nM)	P-value (difference, K562 vs K562-XR)
K562 + imatinib	387* (319–470)		102 (37–284)	
K562 + nilotinib	4* (1–18)		9.4 (3–30)	
K562 + dasatinib	0.3* (0.1–0.5)		32 (8–130)	
K562-IR + imatinib	1330*	N/A**	11294 (0.02-inf)	0.0456
K562-IR + nilotinib	59* (54–65)	N/A**	inf**	0.3327**
K562-IR + dasatinib	0.2* (0.1–0.5)	0.0077	inf**	0.2001**
K562-NR + imatinib	9009	0.2122***	533 (173–1644)	0.0396
K562-NR + nilotinib	718 (202–2544)	<0.0001	21 (5.6–82)	0.4071
K562-NR + dasatinib	3.6 (1.7–7.9)	<0.0001	40 (6.7–233)	0.8449
K562-DR + imatinib	626* (496–791)	<0.0001	508 (248–1043)	0.0221
K562-DR + nilotinib	25 (11–54)	0.0069	21 (6.8–66)	0.3934
K562-DR + dasatinib	2.6* (0.78–8.6)	<0.0001	87 (30–251)	0.2928

IC_50_ values calculated using either three-parameter non-linear regression, except where indicated with *, for which best fit to the data was achieved using four-parameter non-linear regression.

**Some fits were ambiguous and statistical comparisons of IC_50_ curve fits (sum-of-squares F-test), IC_50_ values and shifts could not be reliably calculated.

***In some cases, p-value calculations were affected by the ambiguity of the fit.

Compared to the TKI-sensitive parental cells, all three resistant cell lines exhibited shifts in IC_50_ values for BcrAbl activity inhibition, however as with the growth curve data, statistical analysis of significance was confounded in some cases by the ambiguity of the non-linear regression curve fits and inability to fit both curves to the same model. (Table 1, Fig 5). Even when the fit was ambiguous, significant differences were seen in the degree of cell growth inhibition in the resistant cell lines at the inhibitor concentrations that had been achieved in the resistant cultures vs. vehicle treatment (see alternate presentation of the data from relevant inhibitor concentrations from the IC_50_ experiment in Fig 5). BcrAbl activity inhibition, on the other hand, was not necessarily correlated to cell growth inhibition resistance in these models (Fig 5 and alternate presentation in Fig 6). In K562-IR cells, BcrAbl activity was inhibited at the concentration of imatinib present in the resistant cell line culture conditions (1 μM), despite the continued growth of the cells at that concentration (Fig 6A and 6D). In K562-NR cells, BcrAbl activity was attenuated relative to the parental K562 cell line even in the vehicle control, as well as in the presence of the concentration of nilotinib consistent with the culture conditions (100 nM, Fig 6B and 6E). Dasatinib treatment was different however, with the parental cell line exhibiting no significant BcrAbl activity inhibition in the presence of the level of dasatinib consistent with the resistant cell line culture conditions (10 nM), despite lack of growth of those cells at that concentration (Fig 6C and 6F), and furthermore, the K562-DR cells showed increased BcrAbl activity at baseline, with moderate, but significant, inhibition at the concentration of dasatinib present in the culture conditions.

**Fig 7:**
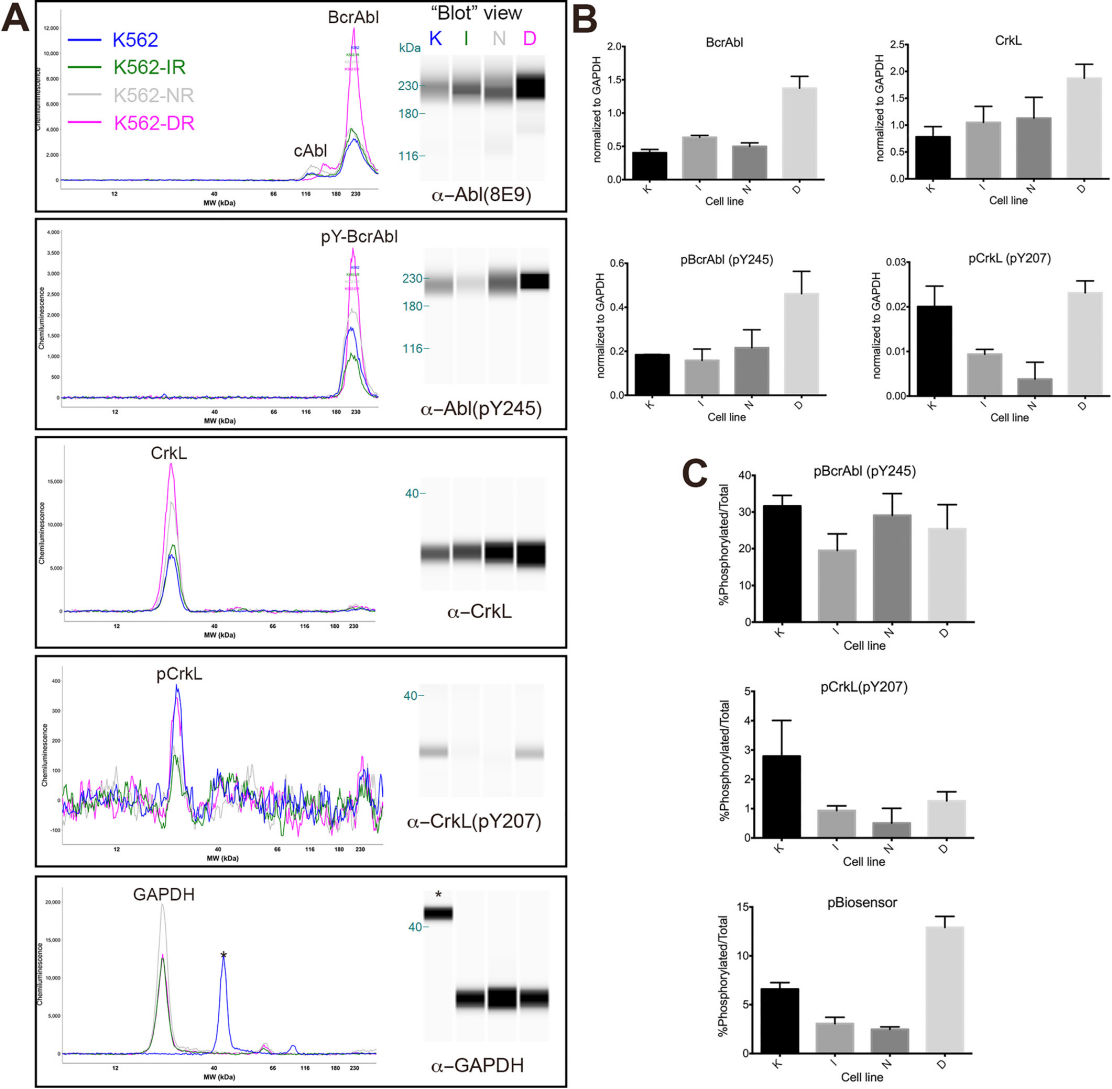
Immunodetection of Abl/BcrAbl, CrkL, and relevant site-specific phosphorylation. Lysates from parental (K), K562-IR (I), K562-NR (N) and K562-DR (D) cells cultured in the absence (K) or presence (I, N and D) of maintenance concentrations of their respective TKIs were analysed in duplicate using the Wes capillary immunodetection system from ProteinSimple. (A) Individual capillary plots and pseudo-“blot” views (in which signal peaks are represented in “band” form) from chemiluminescence for one replicate from each antibody analysis: total Abl/BcrAbl (αAbl #2862, Cell Signaling Technology), phospho-Abl/BcrAbl (αpY245 ab195839, Abcam), total CrkL (αCrkL 32H4/#3182, Cell Signaling Technology), phospho-CrkL (αpY207 EP270Y/ab52908), and GAPDH (αGAPDH D16H11, Cell Signaling Technology). Peaks are labelled for the respective proteins; note that for GAPDH, one capillary lane (labelled with *) in this replicate exhibited artifactually shifted migration, however immunodetection was consistent with other capillaries. (B) Peaks from total BcrAbl, pYBcrAbl, total CrkL and pYCrkL were normalized to peaks from GAPDH for each duplicate, and plotted to show comparisons between the parental (K) and drug-resistant (I, N, D) cells. Error bars indicate SEM for duplicate runs. (C) GAPDH-normalized signals were used to calculate and compare %phosphorylated/total signal for BcrAbl (top) and CrkL (middle). Estimated % phosphorylated biosensor calibrated from the standard curve and compared with total binding capacity of the well, as described in Fig 6 legend) was re-plotted from data shown in Figs 4 and 6, and shown here (bottom) for comparison to endogenous protein %phospho/total. Note that “blot” images are digital representations of peak intensity, *not* photographic or scanned images of actual blots, and thus band shapes appear unnatural.

The lack of correspondence between BcrAbl activity inhibition and the apparent increase in baseline BcrAbl activity in dasatinib-resistant cells were intriguing, but could have resulted from factors specific to the biosensor assay and not necessarily be reflective of endogenous BcrAbl activity. Therefore, it was important to examine endogenous substrate phosphorylation in each cell line as an internal control to validate the interpretation of BcrAbl activity observed by the biosensor assay. Using the Wes platform from ProteinSimple (a capillary-based Western blotting system for immunodetection), we measured protein levels and site-specific phosphorylation for BcrAbl, c-Abl, and CrkL in the parental K562 cell line and in each resistant cell line cultured at the maintenance concentration of its respective inhibitor. Data are summarized in Fig 7. Analysis of protein levels showed that BcrAbl protein was upregulated in the dasatinib-resistant cell line (Fig 7A top and B top left), which is consistent with the increased BcrAbl activity observed in the biosensor assay in those cells. CrkL protein was also moderately upregulated in the dasatinib-resistant cells. Phosphorylated BcrAbl and phosphorylated CrkL were also elevated in dasatinib-resistant cells, consistent with the increased BcrAbl expression, and the increased activity observed in the biosensor assay. Both proteins were at normal levels (relative to the parental cell line control), however, in the imatinib- and nilotinib-resistant cell lines. BcrAbl phosphorylation was also at normal levels in imatinib- and nilotinib-resistant cells, however CrkL phosphorylation was decreased in both, which was also consistent with what was observed in the biosensor assay. These results from endogenous controls validate the interpretation of biosensor phosphorylation as specific to BcrAbl in these cell lines. Furthermore, evaluation of BcrAbl and CrkL phosphorylation as a function of total protein signal for each also highlighted the utility of the biosensor assay to reveal activity differences that could be confounded by changes in expression level for an endogenous target substrate. %pBcrAbl/BcrAbl showed very little difference between cell lines, and %pCrkL/CrkL showed inhibition across all cell lines, because total protein levels had increased in the dasatinib-resistant cells and created a normalization artifact. The biosensor assay, however, reported increased activity as %phosphorylated/total in the dasatinib-resistant cells that likely resulted from the increased expression of the kinase. Because the biosensor is added exogenously, its %phosphorylated/total signal was able to reveal differences in intracellular kinase activity that were not revealed by these commonly used markers for BcrAbl activation.

Overall, these results suggest that resistance to imatinib and nilotinib in these models is likely to be independent of BcrAbl activity (and thus characterized as “off-target”), while resistance to dasatinib may be the result of increased BcrAbl expression (and thus characterized as “on-target”). The specific pathways involved in off-target resistance in our models is not yet known, however we can speculate that they may be related to commonly observed mechanisms, such as increased expression of the ABCG1/ABCG2 efflux pumps, which have been suggested to cause TKI resistance [40,41]. However, this mechanism likely did not solely account for the resistance behavior because BcrAbl activity was still inhibited in cells exhibiting resistant growth at moderate to higher drug concentrations by all three TKIs tested, which are all substrates of these transporters. Other off-target resistance mechanisms, such compensatory upregulation or signalling through alternate pathways may be partially responsible, and have been identified elsewhere with similar TKI resistance models [31,36]. Elucidating the full mechanism of resistance in these cell lines is the subject of ongoing efforts in our laboratory.

Interestingly, examining cross-resistance between TKIs (i.e. resistance to growth or activity inhibition by one inhibitor in a cell line generated by developing resistance to another inhibitor), revealed complex behaviour in the response of resistant cells to next-line drugs (Figs 4 and 5). In imatinib-resistant K562-IR cells, nilotinib and dasatinib were less potent for cell growth inhibition relative to the parental cells, but eventually capable of inhibiting growth at higher concentrations. BcrAbl activity in K562-IR cells, on the other hand, was attenuated relative to parental cells and not effectively inhibited by either nilotinib or dasatinib at concentrations tested (even those at which cell growth had been inhibited). In nilotinib-resistant K562-NR cells, cell growth inhibition by imatinib was dramatically lower (with imatinib unable to inhibit growth at any of the concentrations tested) despite inhibition of BcrAbl activity. Cell growth inhibition was somewhat less potent for dasatinib in K562-NR cells, but not as dramatically as was observed for imatinib, and BcrAbl activity was inhibited. In dasatinib-resistant cells, cell growth inhibition potency for imatinib and nilotinib was only slightly shifted relative to parental cells, and although BcrAbl activity levels were higher at baseline, IC_50_ values were not substantially different from those for parental cells.

Overall, these cross-resistance results suggest that mechanisms of kinase inhibitor resistance in K562 cells are complex, and not necessarily explained by the activity of the primary inhibitor target alone. While we cannot currently speculate on the mechanisms for this cross-resistance behaviour, we are following up to further investigate these in ongoing work that exceeds the scope of this demonstration of the biosensor assay in these model systems. It is important to keep in mind that drug resistance here evolved *in vitro* from a particular cell line, over a shorter amount of time and under much lower drug concentrations than are typically achieved in serum [42] and not *in vivo* or in a patient treated for a native hematopoietic malignancy. Still, these cross-resistance behaviors suggest that response to a particular kinase inhibitor in cells can potentially be affected by resistance to another, regardless of the status of BcrAbl target inhibition by the drug. We stress that this information cannot be used to make predictions or interpretations about treatment choices in the clinic, but it does highlight the potential utility of a cell-based assay such as this one to characterize the target-dependence of drug resistance in a comprehensive study in primary cells in the future.

## Conclusions

In summary, we have demonstrated a multi-well plate compatible, cell-based assay for directly measuring endogenous BcrAbl kinase activity. The conditions were standardized for reasonable throughput and reproducibility, and could be used in a typical format in pre-clinical drug discovery as, for example, a secondary cell-based follow-up protocol for use after preliminary high-throughput screening. Relative to its closest relevant comparison, sandwich ELISA for an endogenous substrate, this assay could be more adaptable and potentially lower cost, based on the reduction in number of antibodies required by eliminating the need for a substrate capture antibody, and on the robust generalizability of capturing substrate via biotin/streptavidin interaction and detecting phosphorylation with the generic anti-phosphotyrosine antibody 4G10, as opposed to needing unique capture and site-specific detection antibodies (which routinely cost >$1000 for production of a lot of antibody, and can cost >$10,000 for development of a novel antibody) for each new substrate to be analysed. The assay accurately reports BcrAbl activity modulated by well-characterized TKIs in both TKI-sensitive and -resistant human chronic myeloid leukemia cells. It also provided information about the complex relationship between BcrAbl activity inhibition and corresponding growth inhibition (i.e. target-dependence). Also, using artificial, multi-functional peptide substrates to measure endogenous kinase activity in disease-relevant cell lines as described in this report may be generalizable to most kinases. Work from our group and others has expanded the repertoire of specific sequences that could be used as cell-penetrating peptide substrates for kinases of interest [21,43–46]. With these and future development of substrates specific for additional kinases, this assay strategy can be further developed with additional disease relevant cell models to identify and characterize TKIs and resistance profiles across the kinome, potentially revealing new options in drug discovery for next-line therapeutic development.

## Supporting Information

S1 Fig Optimization of conditions for high-throughput cell-based BcrAbl activity assay. K562 cells (750,000 cells per well) were exposed to a range of BcrAbl substrate concentrations (n = 8). (A) Coefficient of variation (CV) and (B) signal to noise ratio (SNR) was calculated for each concentration. The results demonstrate that the highest SNR and lowest CV values occur at the lowest peptide concentrations tested.
(TIFF)

S2 Fig Cellular uptake of BcrAbl substrate in K562 cells. The BcrAbl substrate was labeled with AlexaFluor 488 and incubated with K562 cells at the indicated concentrations. Cells were washed with PBS, and cellular uptake was assessed using flow cytometry (10,000 events). Cell distributions shift rightward with increasing substrate concentration, indicating that the cells take up substrate proportionally to the amount they are incubated with. These results suggest that increasing substrate concentration does not disrupt cellular uptake.
(TIFF)

S3 Fig Effect of BcrAbl substrate concentration on localization in K562 cells. The BcrAbl substrate was labeled with AlexaFluor 488 and incubated with K562 cells at the indicated concentrations for 5 minutes. Cells were washed and suspended in CyGel on a slide for fluorescence microscopy. Images for at least two cells were recorded at each concentration. Each pair of images (bright field + fluorescent overlay and fluorescent only) represents a separate, distinct cell. Intensity of cytoplasmically localized peptide was generally higher at higher peptide concentrations, however most cells exhibited punctate localization of peptide, as has been previously observed for this peptide in the HEK293 cell line.
(TIFF)

S4 Fig Concentrations of the BcrAbl substrate in the range used in this study do not affect intracellular BcrAbl activity. Western blots were used to determine if the BcrAbl substrate concentration range used here has an effect on intracellular BcrAbl signaling. An endogenous substrate of BcrAbl, CrkL, was monitored by Western blot in the presence of a range of substrate concentrations. Phosphorylation of CrkL on tyrosine 207 is indicative of intracellular BcrAbl activity. The results show that phosphorylation state of CrkL was not modulated by the BcrAbl substrate.
(TIFF)

S5 Fig LC/MS characterization of BcrAbl substrate peptide used in experiments. Top panel: LC/MS trace of peptide substrate (arrow indicates substrate peptide; Non-natural amino acids: B—biotinylated lysine, J–Photocleavable linker). Middle panel: Relative abundanace of peptide substrate shows purity of peptide peak as most abundant ion with >90% purity. Bottom panel: Expected masses for different charge states of the substrate.
(TIFF)

S6 Fig LC/MS characterization of BcrAbl substrate peptide labeled with Alexa Fluor 488. Top panel: LC/MS trace of peptide substrate (arrow indicates substrate peptide; Non-natural amino acids: B—biotinylated lysine, J–Photocleavable linker, C488 –Alexa Fluor 488 labeled cysteine). Middle panel: Relative abundanace of peptide substrate shows purity of peptide peak as most abundant ion with >90% purity. Bottom panel: Expected masses for different charge states of the substrate.
(TIFF)

S7 Fig LC/MS characterization of the phosphorylated BcrAbl substrate peptide. Top panel: LC/MS trace of peptide substrate (arrow indicates substrate peptide; Non-natural amino acids: B—biotinylated lysine, J–Photocleavable linker, pY–phosphorylated tyrosine). Middle panel: Relative abundanace of peptide substrate shows purity of peptide peak as most abundant ion with >70% purity. Bottom panel: Expected masses for different charge states of the substrate.
(PDF)

S1 File Wes.zip. Supplementary files containing raw data and protocols from immunoblots performed with the Wes Simple Western (ProteinSimple) system.
(ZIP)

S1 Methods Supplementary methods for data in supplemental figures. (PDF)

S1 Table BcrAbl substrate full sequence and functional sequences. B–biotinylated lysine, J–photocleavable linker, C–Cysteine used to label with Alexa Fluor 488
(TIFF)

## References

[1] Zhang J, Yang PL, Gray NS. Targeting cancer with small molecule kinase inhibitors. Nature reviews Cancer. 2009;9(1):28–39. Epub 2008/12/24. 10.1038/nrc2559 . 19104514 PMC12406740

[2] Jabbour E, Kantarjian H. Chronic myeloid leukemia: 2014 update on diagnosis, monitoring, and management. American journal of hematology. 2014;89(5):547–56. Epub 2014/04/15. 10.1002/ajh.23691 . 24729196

[3] Druker BJ, Guilhot F, O'Brien SG, Gathmann I, Kantarjian H, Gattermann N, et al Five-year follow-up of patients receiving imatinib for chronic myeloid leukemia. The New England journal of medicine. 2006;355(23):2408–17. Epub 2006/12/08. 10.1056/NEJMoa062867 . 17151364

[4] Saglio G, Kim DW, Issaragrisil S, le Coutre P, Etienne G, Lobo C, et al Nilotinib versus imatinib for newly diagnosed chronic myeloid leukemia. The New England journal of medicine. 2010;362(24):2251–9. Epub 2010/06/08. 10.1056/NEJMoa0912614 . 20525993

[5] Kantarjian H, Shah NP, Hochhaus A, Cortes J, Shah S, Ayala M, et al Dasatinib versus imatinib in newly diagnosed chronic-phase chronic myeloid leukemia. The New England journal of medicine. 2010;362(24):2260–70. Epub 2010/06/08. 10.1056/NEJMoa1002315 . 20525995

[6] Brummendorf TH, Cortes JE, de Souza CA, Guilhot F, Duvillie L, Pavlov D, et al Bosutinib versus imatinib in newly diagnosed chronic-phase chronic myeloid leukaemia: results from the 24-month follow-up of the BELA trial. British journal of haematology. 2015;168(1):69–81. Epub 2014/09/10. 10.1111/bjh.13108 ; PubMed Central PMCID: PMCPmc4274978. 25196702 PMC4274978

[7] Cortes JE, Kantarjian H, Shah NP, Bixby D, Mauro MJ, Flinn I, et al Ponatinib in refractory Philadelphia chromosome-positive leukemias. The New England journal of medicine. 2012;367(22):2075–88. Epub 2012/11/30. 10.1056/NEJMoa1205127 ; PubMed Central PMCID: PMCPmc3777383. 23190221 PMC3777383

[8] Kim SH, Menon H, Jootar S, Saikia T, Kwak JY, Sohn SK, et al Efficacy and safety of radotinib in chronic phase chronic myeloid leukemia patients with resistance or intolerance to BCR-ABL1 tyrosine kinase inhibitors. Haematologica. 2014;99(7):1191–6. Epub 2014/04/08. 10.3324/haematol.2013.096776 ; PubMed Central PMCID: PMCPmc4077080. 24705186 PMC4077080

[9] Pan X, Dong J, Shi Y, Shao R, Wei F, Wang J, et al Discovery of novel Bcr-Abl inhibitors with diacylated piperazine as the flexible linker. Organic & biomolecular chemistry. 2015;13(25):7050–66. Epub 2015/06/09. 10.1039/c5ob00430f . 26052668

[10] Warmuth M, Kim S, Gu XJ, Xia G, Adrian F. Ba/F3 cells and their use in kinase drug discovery. Current opinion in oncology. 2007;19(1):55–60. Epub 2006/11/30. 10.1097/CCO.0b013e328011a25f . 17133113

[11] Drew AE, Al-Assaad S, Yu V, Andrews P, Merkel P, Szilvassy S, et al Comparison of 2 cell-based phosphoprotein assays to support screening and development of an ALK inhibitor. Journal of biomolecular screening. 2011;16(2):164–73. Epub 2011/02/08. 10.1177/1087057110394657 . 21297104

[12] Smith GKW E. R. Cell-based assays for kinase drug discovery. Drug Discovery Today: Technologies. 2010;7(1):e13–e9. Epub 2010/04/01. 10.1016/j.ddtec.2010.04.002 . 24103680

[13] White D, Saunders V, Grigg A, Arthur C, Filshie R, Leahy MF, et al Measurement of in vivo BCR-ABL kinase inhibition to monitor imatinib-induced target blockade and predict response in chronic myeloid leukemia. Journal of clinical oncology: official journal of the American Society of Clinical Oncology. 2007;25(28):4445–51. Epub 2007/10/02. 10.1200/jco.2006.09.9499 . 17906206

[14] Folkvord S, Flatmark K, Dueland S, de Wijn R, Groholt KK, Hole KH, et al Prediction of response to preoperative chemoradiotherapy in rectal cancer by multiplex kinase activity profiling. International journal of radiation oncology, biology, physics. 2010;78(2):555–62. Epub 2010/08/03. 10.1016/j.ijrobp.2010.04.036 . 20675069

[15] Peled N, Wynes MW, Ikeda N, Ohira T, Yoshida K, Qian J, et al Insulin-like growth factor-1 receptor (IGF-1R) as a biomarker for resistance to the tyrosine kinase inhibitor gefitinib in non-small cell lung cancer. Cellular oncology (Dordrecht). 2013;36(4):277–88. Epub 2013/04/27. 10.1007/s13402-013-0133-9 ; PubMed Central PMCID: PMCPmc4186686. 23619944 PMC4186686

[16] Boros K, Puissant A, Back M, Alexe G, Bassil CF, Sinha P, et al Increased SYK activity is associated with unfavorable outcome among patients with acute myeloid leukemia. Oncotarget. 2015;6(28):25575–87. Epub 2015/09/01. 10.18632/oncotarget.4669 . 26315286 PMC4694851

[17] Eglen RM, Reisine T, Roby P, Rouleau N, Illy C, Bosse R, et al The use of AlphaScreen technology in HTS: current status. Current chemical genomics. 2008;1:2–10. Epub 2008/01/01. 10.2174/1875397300801010002 ; PubMed Central PMCID: PMCPmc2775125. 20161822 PMC2775125

[18] Smith AL, DeMorin FF, Paras NA, Huang Q, Petkus JK, Doherty EM, et al Selective inhibitors of the mutant B-Raf pathway: discovery of a potent and orally bioavailable aminoisoquinoline. Journal of medicinal chemistry. 2009;52(20):6189–92. Epub 2009/09/22. 10.1021/jm901081g . 19764794

[19] Wittman MD, Carboni JM, Yang Z, Lee FY, Antman M, Attar R, et al Discovery of a 2,4-disubstituted pyrrolo[1,2-f][1,2,4]triazine inhibitor (BMS-754807) of insulin-like growth factor receptor (IGF-1R) kinase in clinical development. Journal of medicinal chemistry. 2009;52(23):7360–3. Epub 2009/09/26. 10.1021/jm900786r . 19778024

[20] Lu L, Ghose AK, Quail MR, Albom MS, Durkin JT, Holskin BP, et al ALK mutants in the kinase domain exhibit altered kinase activity and differential sensitivity to small molecule ALK inhibitors. Biochemistry. 2009;48(16):3600–9. Epub 2009/03/03. 10.1021/bi8020923 . 19249873

[21] Lipchik AM, Killins RL, Geahlen RL, Parker LL. A peptide-based biosensor assay to detect intracellular Syk kinase activation and inhibition. Biochemistry. 2012;51(38):7515–24. Epub 2012/08/28. 10.1021/bi300970h ; PubMed Central PMCID: PMCPmc3545090. 22920457 PMC3545090

[22] Frietsch JJ, Kastner C, Grunewald TG, Schweigel H, Nollau P, Ziermann J, et al LASP1 is a novel BCR-ABL substrate and a phosphorylation-dependent binding partner of CRKL in chronic myeloid leukemia. Oncotarget. 2014;5(14):5257–71. Epub 2014/06/11. 10.18632/oncotarget.2072 ; PubMed Central PMCID: PMCPmc4170624. 24913448 PMC4170624

[23] Morris MC. Fluorescent biosensors—probing protein kinase function in cancer and drug discovery. Biochimica et biophysica acta. 2013;1834(7):1387–95. Epub 2013/02/05. 10.1016/j.bbapap.2013.01.025 . 23376184

[24] Mizutani T, Kondo T, Darmanin S, Tsuda M, Tanaka S, Tobiume M, et al A novel FRET-based biosensor for the measurement of BCR-ABL activity and its response to drugs in living cells. Clinical cancer research: an official journal of the American Association for Cancer Research. 2010;16(15):3964–75. Epub 2010/07/31. 10.1158/1078-0432.ccr-10-0548 . 20670950

[25] Mathrubutham MF, W; Shankaran, N; Ercken, M; Wehrman, T; Olson, K. R. Application of the PathHunter™ Protein Interaction Assay to Receptor Tyrosine Kinases (RTKs): Developing a Non-Antibody One-Step Cell-Based Kinase Activity. Proceedings of the 15th Annual Society for Biomolecular Sciences Conference. 2009.

[26] Hsu J, Zhang J, Kitson C, Tan SL, Narula S, DeMartino JA, et al Development of a pharmacodynamic assay based on PLCgamma2 phosphorylation for quantifying spleen tyrosine kinase (SYK)-Bruton's tyrosine kinase (BTK) signaling. Journal of biomolecular screening. 2013;18(8):890–8. Epub 2013/05/25. 10.1177/1087057113489881 . 23704133

[27] Placzek EA, Plebanek MP, Lipchik AM, Kidd SR, Parker LL. A peptide biosensor for detecting intracellular Abl kinase activity using matrix-assisted laser desorption/ionization time-of-flight mass spectrometry. Analytical biochemistry. 2010;397(1):73–8. Epub 2009/10/13. 10.1016/j.ab.2009.09.048 ; PubMed Central PMCID: PMCPmc2808441. 19818327 PMC2808441

[28] Tang J, Wang JY, Parker LL. Detection of early Abl kinase activation after ionizing radiation by using a peptide biosensor. Chembiochem: a European journal of chemical biology. 2012;13(5):665–73. Epub 2012/02/16. 10.1002/cbic.201100763 ; PubMed Central PMCID: PMCPmc3429332. 22334513 PMC3429332

[29] Yang TY, Eissler CL, Hall MC, Parker LL. A multiple reaction monitoring (MRM) method to detect Bcr-Abl kinase activity in CML using a peptide biosensor. PLOS ONE. 2013;8(2):e56627 Epub 2013/02/26. 10.1371/journal.pone.0056627 ; PubMed Central PMCID: PMCPmc3577862. 23437189 PMC3577862

[30] Mahon FX, Deininger MW, Schultheis B, Chabrol J, Reiffers J, Goldman JM, et al Selection and characterization of BCR-ABL positive cell lines with differential sensitivity to the tyrosine kinase inhibitor STI571: diverse mechanisms of resistance. Blood. 2000;96(3):1070–9. Epub 2000/07/27. . 10910924

[31] Gioia R, Leroy C, Drullion C, Lagarde V, Etienne G, Dulucq S, et al Quantitative phosphoproteomics revealed interplay between Syk and Lyn in the resistance to nilotinib in chronic myeloid leukemia cells. Blood. 2011;118(8):2211–21. Epub 2011/07/07. 10.1182/blood-2010-10-313692 . 21730355

[32] Iversen PW, Beck B, Chen YF, Dere W, Devanarayan V, Eastwood BJ, et al HTS Assay Validation In: Sittampalam GS, Gal-Edd N, Arkin M, Auld D, Austin C, Bejcek B, et al, editors. Assay Guidance Manual. Bethesda (MD): Eli Lilly & Company and the National Center for Advancing Translational Sciences; 2004. 22553862

[33] Mand MR, Wu D, Veach DR, Kron SJ. Cell treatment and lysis in 96-well filter-bottom plates for screening Bcr-Abl activity and inhibition in whole-cell extracts. Journal of biomolecular screening. 2010;15(4):434–40. Epub 2010/03/20. 10.1177/1087057110363307 . 20237206 PMC4471859

[34] Zhang JH, Chung TD, Oldenburg KR. A Simple Statistical Parameter for Use in Evaluation and Validation of High Throughput Screening Assays. Journal of biomolecular screening. 1999;4(2):67–73. Epub 2000/06/06. . 10838414 10.1177/108705719900400206

[35] Kitagawa D, Yokota K, Gouda M, Narumi Y, Ohmoto H, Nishiwaki E, et al Activity-based kinase profiling of approved tyrosine kinase inhibitors. Genes to cells: devoted to molecular & cellular mechanisms. 2013;18(2):110–22. Epub 2013/01/03. 10.1111/gtc.12022 . 23279183

[36] Dufies M, Jacquel A, Belhacene N, Robert G, Cluzeau T, Luciano F, et al Mechanisms of AXL overexpression and function in Imatinib-resistant chronic myeloid leukemia cells. Oncotarget. 2011;2(11):874–85. Epub 2011/12/06. ; PubMed Central PMCID: PMCPmc3259992. 22141136 10.18632/oncotarget.360 PMC3259992

[37] Cassuto O, Dufies M, Jacquel A, Robert G, Ginet C, Dubois A, et al All tyrosine kinase inhibitor-resistant chronic myelogenous cells are highly sensitive to ponatinib. Oncotarget. 2012;3(12):1557–65. Epub 2012/12/15. ; PubMed Central PMCID: PMCPmc3681494. 23238683 10.18632/oncotarget.692 PMC3681494

[38] Okabe S, Tauchi T, Tanaka Y, Ohyashiki K. Dasatinib preferentially induces apoptosis by inhibiting Lyn kinase in nilotinib-resistant chronic myeloid leukemia cell line. Journal of hematology & oncology. 2011;4(1):32 Epub 2011/08/03. 10.1186/1756-8722-4-32 ; PubMed Central PMCID: PMCPmc3163636. 21806844 PMC3163636

[39] Tang C, Schafranek L, Watkins DB, Parker WT, Moore S, Prime JA, et al Tyrosine kinase inhibitor resistance in chronic myeloid leukemia cell lines: investigating resistance pathways. Leukemia & lymphoma. 2011;52(11):2139–47. Epub 2011/07/02. 10.3109/10428194.2011.591013 . 21718141

[40] Eadie LN, Hughes TP, White DL. Interaction of the efflux transporters ABCB1 and ABCG2 with imatinib, nilotinib, and dasatinib. Clinical pharmacology and therapeutics. 2014;95(3):294–306. Epub 2013/10/11. 10.1038/clpt.2013.208 . 24107928

[41] Kim YK, Lee SS, Jeong SH, Ahn JS, Yang DH, Lee JJ, et al OCT-1, ABCB1, and ABCG2 Expression in Imatinib-Resistant Chronic Myeloid Leukemia Treated with Dasatinib or Nilotinib. Chonnam medical journal. 2014;50(3):102–11. Epub 2015/01/09. 10.4068/cmj.2014.50.3.102 ; PubMed Central PMCID: PMCPmc4276791. 25568846 PMC4276791

[42] Rezende VM, Rivellis AJ, Gomes MM, Dorr FA, Novaes MM, Nardinelli L, et al Determination of serum levels of imatinib mesylate in patients with chronic myeloid leukemia: validation and application of a new analytical method to monitor treatment compliance. Revista brasileira de hematologia e hemoterapia. 2013;35(2):103–8. Epub 2013/06/07. 10.5581/1516-8484.20130030 ; PubMed Central PMCID: PMCPmc3672119. 23741187 PMC3672119

[43] Cui W, Parker LL. A time-resolved luminescence biosensor assay for anaplastic lymphoma kinase (ALK) activity. Chemical communications (Cambridge, England). 2015;51(2):362–5. Epub 2014/11/20. 10.1039/c4cc07453j ; PubMed Central PMCID: PMCPmc4288484. 25406835 PMC4288484

[44] Lipchik AM, Perez M, Bolton S, Dumrongprechachan V, Ouellette SB, Cui W, et al KINATEST-ID: a pipeline to develop phosphorylation-dependent terbium sensitizing kinase assays. Journal of the American Chemical Society. 2015;137(7):2484–94. Epub 2015/02/18. 10.1021/ja507164a ; PubMed Central PMCID: PMCPmc4342272. 25689372 PMC4342272

[45] Li H, Sims CE, Kaluzova M, Stanbridge EJ, Allbritton NL. A quantitative single-cell assay for protein kinase B reveals important insights into the biochemical behavior of an intracellular substrate peptide. Biochemistry. 2004;43(6):1599–608. Epub 2004/02/11. 10.1021/bi035597k . 14769036

[46] Xu W, Allbritton N, Lawrence DS. SRC kinase regulation in progressively invasive cancer. PLOS ONE. 2012;7(11):e48867 Epub 2012/11/13. 10.1371/journal.pone.0048867 23145001 PMC3492248

