# Supplementary figures and images for: A Cell-Based Assay for Measuring Endogenous BcrAbl Kinase Activity and Inhibitor Resistance
Source: PLoS One. 2016 Sep 6;11(9):e0161748. doi: 10.1371/journal.pone.0161748 (PMC5012566; doi:10.1371/journal.pone.0161748)

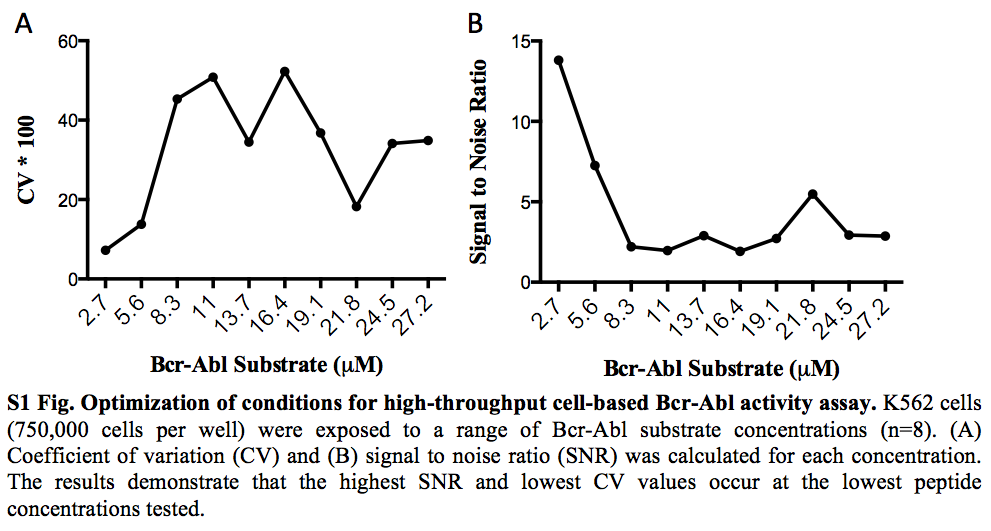

Supplement: S1 Fig — K562 cells (750,000 cells per well) were exposed to a range of BcrAbl substrate concentrations (n = 8). (A) Coefficient of variation (CV) and (B) signal to noise ratio (SNR) was calculated for each concentration. The results demonstrate that the highest SNR and lowest CV values occur at the lowest peptide concentrations tested. (TIFF) [file pone.0161748.s001.tiff]

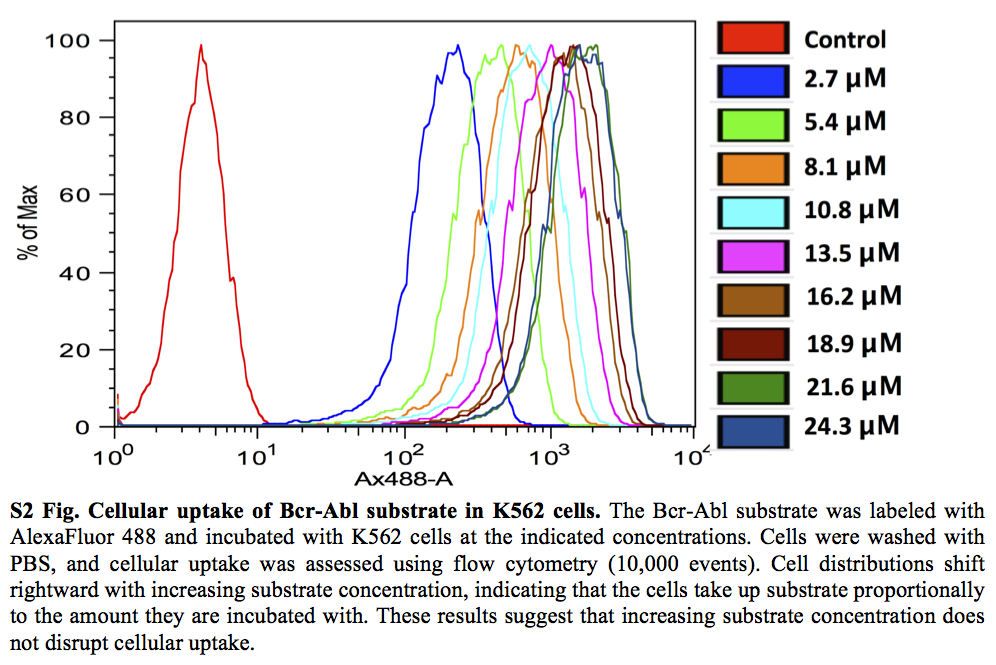

Supplement: S2 Fig — The BcrAbl substrate was labeled with AlexaFluor 488 and incubated with K562 cells at the indicated concentrations. Cells were washed with PBS, and cellular uptake was assessed using flow cytometry (10,000 events). Cell distributions shift rightward with increasing substrate concentration, indicating that the cells take up substrate proportionally to the amount they are incubated with. These results suggest that increasing substrate concentration does not disrupt cellular uptake. (TIFF) [file pone.0161748.s002.tiff]

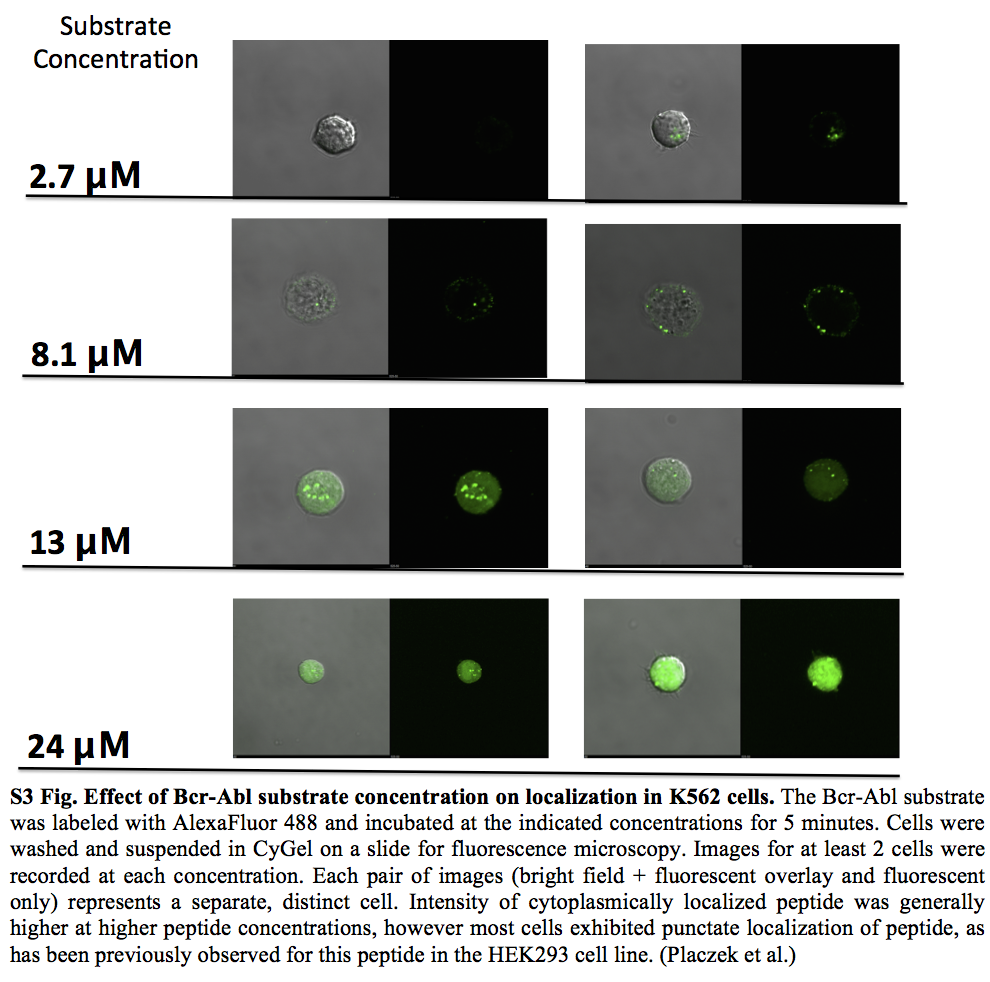

Supplement: S3 Fig — The BcrAbl substrate was labeled with AlexaFluor 488 and incubated with K562 cells at the indicated concentrations for 5 minutes. Cells were washed and suspended in CyGel on a slide for fluorescence microscopy. Images for at least two cells were recorded at each concentration. Each pair of images (bright field + fluorescent overlay and fluorescent only) represents a separate, distinct cell. Intensity of cytoplasmically localized peptide was generally higher at higher peptide concentrations, however most cells exhibited punctate localization of peptide, as has been previously observed for this peptide in the HEK293 cell line. (TIFF) [file pone.0161748.s003.tiff]

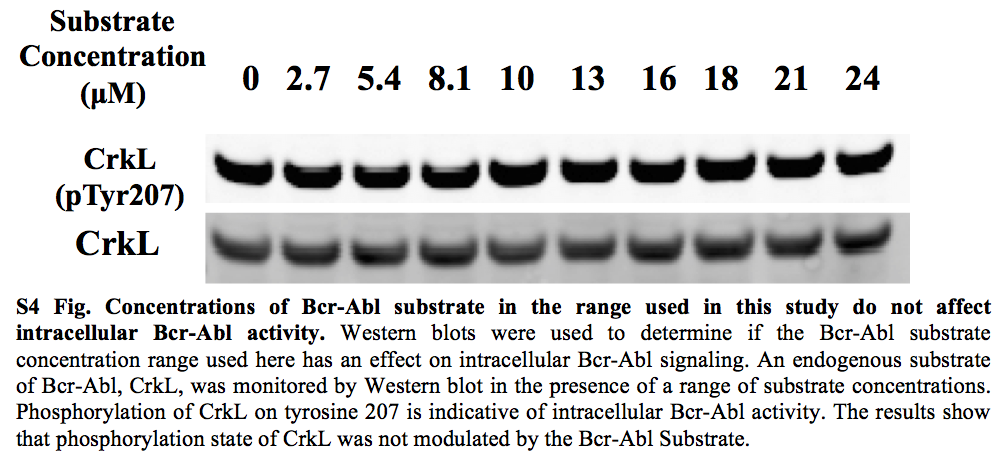

Supplement: S4 Fig — Western blots were used to determine if the BcrAbl substrate concentration range used here has an effect on intracellular BcrAbl signaling. An endogenous substrate of BcrAbl, CrkL, was monitored by Western blot in the presence of a range of substrate concentrations. Phosphorylation of CrkL on tyrosine 207 is indicative of intracellular BcrAbl activity. The results show that phosphorylation state of CrkL was not modulated by the BcrAbl substrate. (TIFF) [file pone.0161748.s004.tiff]

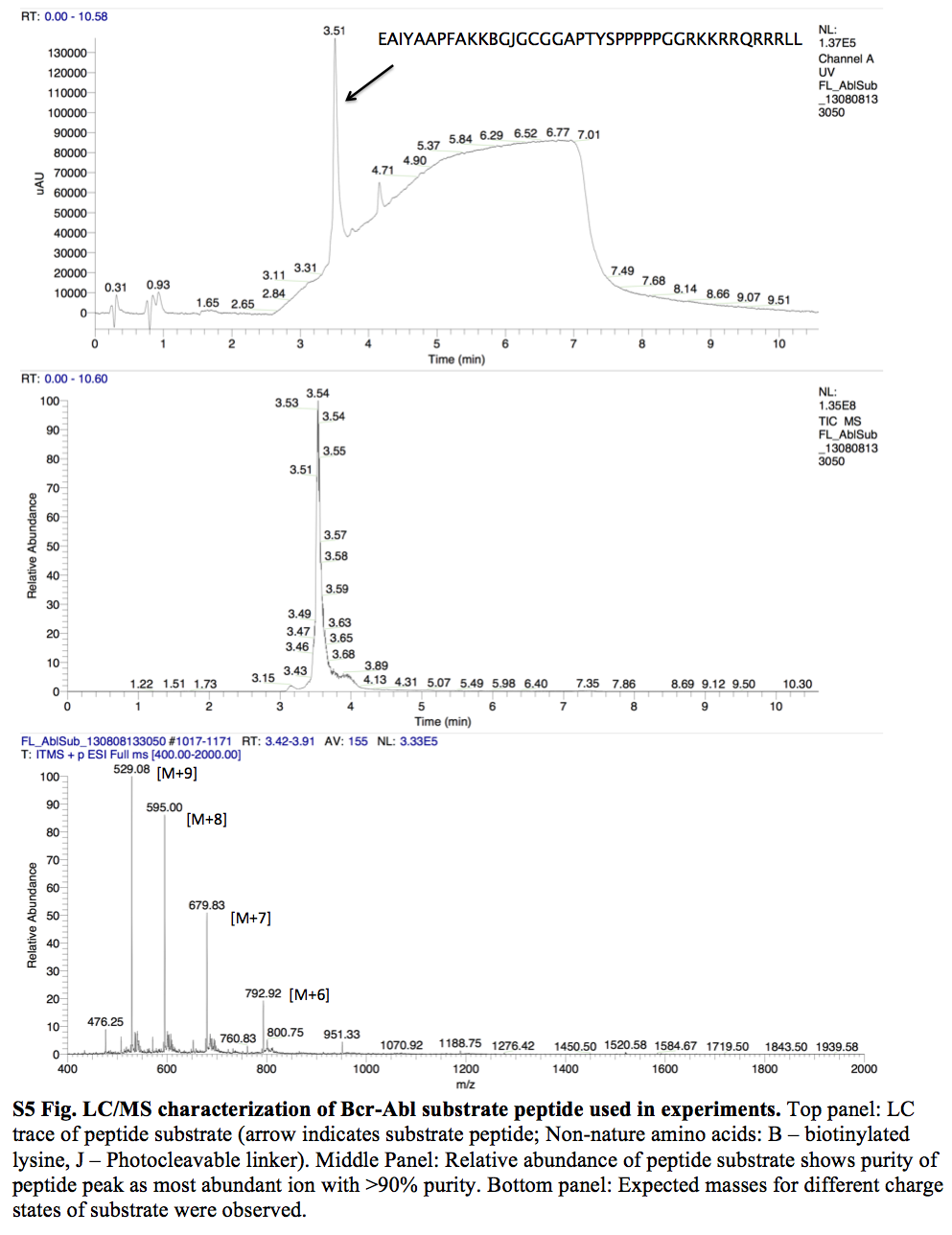

Supplement: S5 Fig — Top panel: LC/MS trace of peptide substrate (arrow indicates substrate peptide; Non-natural amino acids: B—biotinylated lysine, J–Photocleavable linker). Middle panel: Relative abundanace of peptide substrate shows purity of peptide peak as most abundant ion with >90% purity. Bottom panel: Expected masses for different charge states of the substrate. (TIFF) [file pone.0161748.s005.tiff]

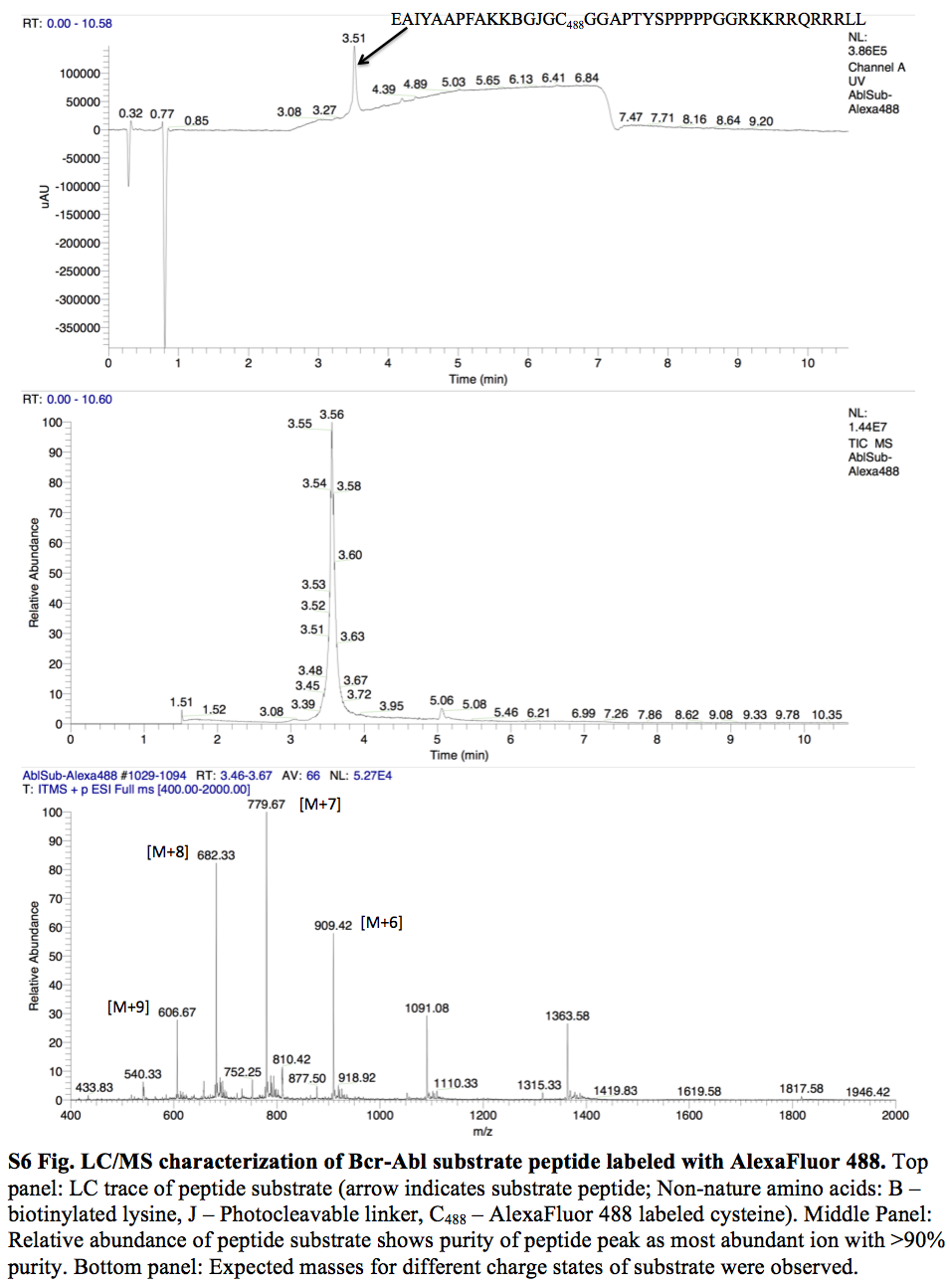

Supplement: S6 Fig — Top panel: LC/MS trace of peptide substrate (arrow indicates substrate peptide; Non-natural amino acids: B—biotinylated lysine, J–Photocleavable linker, C488 –Alexa Fluor 488 labeled cysteine). Middle panel: Relative abundanace of peptide substrate shows purity of peptide peak as most abundant ion with >90% purity. Bottom panel: Expected masses for different charge states of the substrate. (TIFF) [file pone.0161748.s006.tiff]

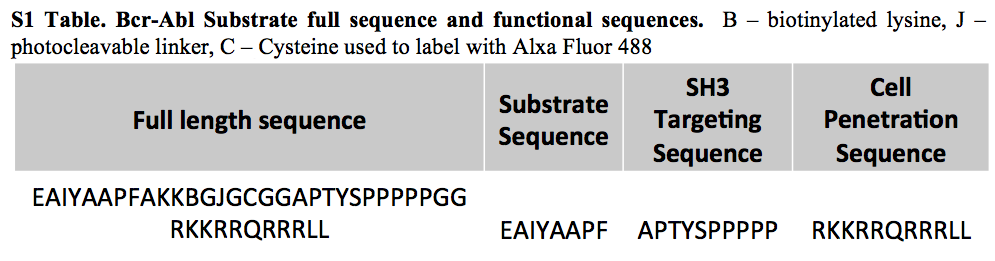

Supplement: S1 Table — B–biotinylated lysine, J–photocleavable linker, C–Cysteine used to label with Alexa Fluor 488 (TIFF) [file pone.0161748.s010.tiff]
