# Supplementary material for: A Cell-Based Assay for Measuring Endogenous BcrAbl Kinase Activity and Inhibitor Resistance
Source: PLoS One. 2016 Sep 6;11(9):e0161748. doi: 10.1371/journal.pone.0161748 (PMC5012566; doi:10.1371/journal.pone.0161748)
Supplement: S7 Fig — Top panel: LC/MS trace of peptide substrate (arrow indicates substrate peptide; Non-natural amino acids: B—biotinylated lysine, J–Photocleavable linker, pY–phosphorylated tyrosine). Middle panel: Relative abundanace of peptide substrate shows purity of peptide peak as most abundant ion with >70% purity. Bottom panel: Expected masses for different charge states of the substrate. (PDF) [file pone.0161748.s007.pdf]

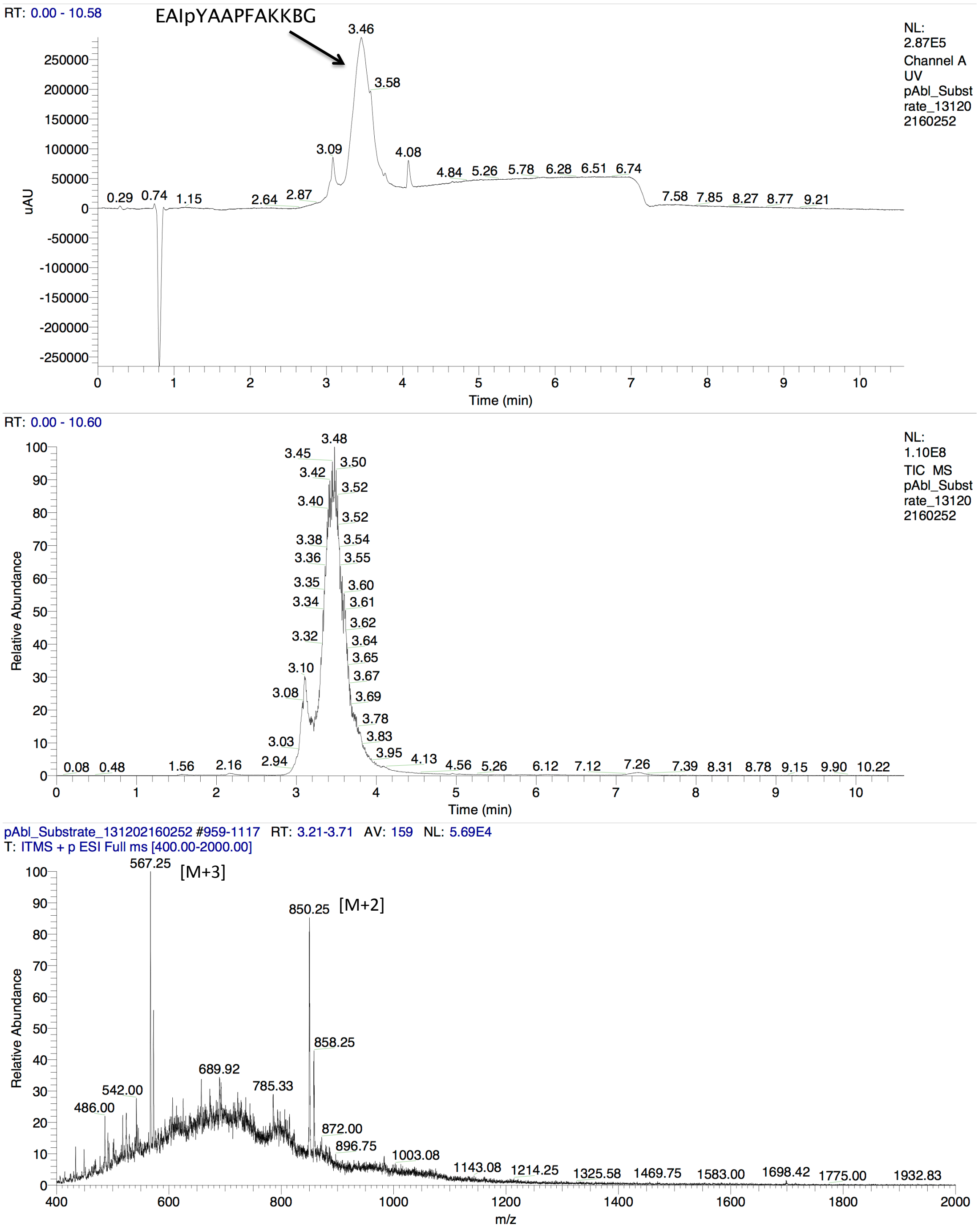

S7 Fig. Top panel: LC trace of peptide substrate (arrow indicates substrate peptide; Non-nature amino acids: B – biotinylated lysine, J – Photocleavable linker, pY – phosphorylated tyrosine). Middle Panel: Relative abundance of peptide substrate shows purity of peptide peak as most abundant ion with >70% purity. Bottom panel: Expected masses for different charge states of substrate were observed.
