# Supplementary material for: A Cell-Based Assay for Measuring Endogenous BcrAbl Kinase Activity and Inhibitor Resistance
Source: PLoS One. 2016 Sep 6;11(9):e0161748. doi: 10.1371/journal.pone.0161748 (PMC5012566; doi:10.1371/journal.pone.0161748)
Supplement: S1 Methods — (PDF) [file pone.0161748.s009.pdf]

Supplementary Methods

Flow cytometry. K562 cells were treated as indicated, and analyzed on a FACSAria III Cell Sorter (BD Biosciences) using the 488 nm laser. A total of 10,000 events were recorded with nonviable cells excluded. Peptide uptake was assessed by the change in mean fluorescent intensity compared to untreated control.

Fluorescent microscopy. K562 cells were treated as indicated and suspended in CyGelTM (Abcam, Cambridge, MA) on glass slides. Fluorescence images where collected on a Nikon A1R confocal microscope using a 40x 1.3 NA oil objective. The dye was excited using a 488 nm laser line and emissions collected from 500-550 nm. Transmitted light DIC images were collected simultaneously.

Immunoblots. Cells were treated as described and pelleted by centrifugation. Media was removed, and cells were washed in ice cold PBS. After washing, lysis buffer (PhosphoSafe Extraction Buffer supplemented with 4 mM EDTA and Complete protease inhibitor (Roche)) was added to the cell pellet and cells were snap frozen in liquid nitrogen. Cell pellets were thawed and vortexed, then centrifuged at 4° C to remove insoluble cellular debris and lysate was assayed for protein content. 70-100 µg of protein was mixed with NuPAGE Laemmli protein gel loading buffer (Invitrogen) and incubated at 95° C for 5 minutes. Lysate were loaded onto a 4-12% Bis-Tris NuPAGE gel (Invitrogen) and separated at constant 160 V for 1.5 hours. Proteins were transferred to a nitrocellulose membrane (Bio-Rad) and blocked with 5% milk in TBST (tris-buffered saline supplemented supplemented with 0.05% Tween-20). Primary antibodies (anti-pY207 CrkL and anti-CrkL, Cell Signaling) were incubated with membrane overnight at 4° C diluted 1:1000 in 5% milk in TBST. Following 3 x 5 minute washes with TBST, the membrane was incubated with goat anti-rabbit tagged with IRDye 680 (1:5000 in TBST + 5% milk) for 2 hours at room temperature. After 3 x 5 minute washes, the membrane was developed on a LI-COR Odyssey infrared scanner.

Quality control analysis peptides. Peptide analysis was performed using a Thermo Accela LC coupled to a Finnigan LTQ mass spectrometer. LC was performed on a Phenomenex C18 column (1.9 µm, 2.1 mm ID x 50 mm) using a 5-65% Solvent B (Acetonitrile, 0.1% Formic Acid), 30%/minute gradient. Mass spectrometry was performed in single MS full scan mode.

Immunodetection using ProteinSimple "Wes" capillary electrophoresis Western blotting system. The method used to analyze K562 and resistant cell lines using the Wes system is described in the materials and methods. Shown here are the raw data exported for analysis with each antibody as both "Chromatogram" (peak) view and "Gel" view. **Please note that the "Gel" view is simply a top-down two-dimensional representation of the peaks shown in the "Chromatogram" view, not an actual Western blot. Because of this, the band shapes are sometimes unnatural, since they are digital representations of peak intensity and not actual photographic or scanned images of bands.
